# Corruption of the Intra-Gene DNA Methylation Architecture Is a Hallmark of Cancer

**DOI:** 10.1371/journal.pone.0068285

**Published:** 2013-07-16

**Authors:** Thomas E. Bartlett, Alexey Zaikin, Sofia C. Olhede, James West, Andrew E. Teschendorff, Martin Widschwendter

**Affiliations:** 1 CoMPLEX, University College London, London, United Kingdom; 2 Deparment of Mathematics, University College London, London, United Kingdom; 3 Deparment of Statistical Science, University College London, London, United Kingdom; 4 Statistical Genomics Group, University College London Cancer Institute, London, United Kingdom; 5 Department of Womens Cancer, University College London Elizabeth Garrett Anderson Institute for Womens Health, London, United Kingdom; Innsbruck Medical University, Austria

## Abstract

Epigenetic processes - including DNA methylation - are increasingly seen as having a fundamental role in chronic diseases like cancer. It is well known that methylation levels at particular genes or loci differ between normal and diseased tissue. Here we investigate whether the intra-gene methylation architecture is corrupted in cancer and whether the variability of levels of methylation of individual CpGs within a defined gene is able to discriminate cancerous from normal tissue, and is associated with heterogeneous tumour phenotype, as defined by gene expression. We analysed 270985 CpGs annotated to 18272 genes, in 3284 cancerous and 681 normal samples, corresponding to 14 different cancer types. In doing so, we found novel differences in intra-gene methylation pattern across phenotypes, particularly in those genes which are crucial for stem cell biology; our measures of intra-gene methylation architecture are a better determinant of phenotype than measures based on mean methylation level alone (K-S test 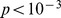 in all 14 diseases tested). These per-gene methylation measures also represent a considerable reduction in complexity, compared to conventional per-CpG beta-values. Our findings strongly support the view that intra-gene methylation architecture has great clinical potential for the development of DNA-based cancer biomarkers.

## Introduction

Epigenetic information is stored in the genome in the form of heritable modifications to the chemical structure of DNA, such as methylation of particular bases, as well a variety of chemical modifications of the histone proteins which package the DNA. Epigenetic information can be modulated during the lifetime of an organism by environmental cues [1]–[3] and these changes persist in subsequent mitoses, leading to an acquired change of phenotype.

DNA methylation is an epigenetic mark consisting almost entirely of the methylation of CpG dinucleotides [4], and it is possible for one, both, or neither alleles at a particular genomic locus to be methylated [5]. Hypermethylation of CpGs in the gene promoter (the region close to the transcriptional start site, TSS) are incontrovertibly associated with silencing of the corresponding gene, and this effect is particularly important in cancer, where aberrant gene silencing is associated with functional changes important in every stage of tumour progression [6].

It has been found previously that variability of methylation at specific genomic locations is important in the development of cancer [7]. It has been noted in particular that there is an increase in stochastic methylation variability in regions which are already known to have altered levels of methylation in cancers, leading to aberrant and varying gene expression, and providing an epigenetic mechanism for tumour heterogeneity [8]. It has also been shown recently that statistics based on differential variability of methylation can lead to improved detection of risk markers in pre-cancerous growths [9], [10].

Polycomb group proteins (PcG) play a fundamental role in developmental processes, maintaining a class of genes known as polycomb group targets (PCGTs) in a repressed state in ES (embryonic stem) cells, to maintain pluripotency, and ‘poised for activation’ during differentiation [11]. The link between PCGTs and cancer has been discussed by many authors [12]–[14]; it was recently shown that DNA hypermethylation in cancers preferentially targets PCGTs which are developmental regulators [15], those authors hypothesising that this may contribute to the stem-like characteristics of cancer; in further support of these ideas it has been noted that tumours which are particularly poorly differentiated tend to display expression patterns which are similar to ES cells, including repression of PCGTs [16].

Polycomb group proteins maintain the repressed state of genes via chromatin (the DNA packaging); DNA in its compact state is wrapped around histone proteins (a main component of chromatin), and PRC2 (polycomb repressive complex 2) is responsible for the trimethylation of lysine 27 of histone 3 (leading to the epigenetic mark H3K27me3), which is associated with this compact state [17]. Genes occupied by PRC2 in ES cells mostly carry bivalent chromatin marks [15]; bivalency includes the histone modification H3K4me3 (trimethylation of lysine 4 on histone 3), a mark which is associated with activation of the corresponding gene, in addition to the repressive H3K27me3 mark. It is thought that it is this bivalent state which maintains stemness, keeping the gene repressed, but poised for activation upon differentiation. As DNA methylation is also associated with repression and activation of genes, it is of interest whether the methylation patterns of genes which carry the chromatin markings H3K27me3 and/or H3K4me3 in stem cells are altered in cancer, as such aberrant alteration of gene regulation via DNA methylation might be associated with a return of or accentuation of stem-like cell characteristics.

The role of early epigenetic changes in oncogenic transformation, including disruption of the healthy epigenotype of progenitor cells, the creation of an epigenetically permissible environment in which genetic aberrations can have tumorigenic effects, and phenotypic plasticity leading to tumour adaptation and associated with intra-tumour heterogeneity, was originally proposed by Feinberg and colleagues [1]. It is hypothesised that one way in which stochastic dysregulation of stem cell genes and associated phenotypic heterogeneity might manifest, is in terms of cell to cell variability of methylation; this would in turn be expected to correlate with intra-gene variability of methylation, as measured using aggregated mixtures of heterogenous cells in a microarray experiment.

Intra-gene methylation variability is deemed to be a disruption of the normal methylation profile, or architecture, of a particular gene, and such a change may be more generally linked to the creation of an epigenetically permissible environment for oncogenic transformation, and to tumourigenesis. Such changes would be expected to accompany the early stages or even precede the onset of the disease, and hence identifying reliable indicators of such changes might provide a valuable lead for the development of DNA-based cancer biomarkers in bodily fluids, especially as it has been shown recently that DNA methylation biomarkers related to stem cell genes are associated with clinical outcome in women’s cancers [18].

Previous studies [7], [9], [10] have focussed on the effects of sample to sample variability of methylation; here for the first time, we analyse the association of phenotype with intra-gene variability of methylation. Making use of data derived from the Illumina Infinium HumanMethylation450 platform, which interrogates 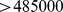 CpGs genome-wide including 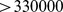 with known gene annotations (corresponding to on average 17 CpGs per gene), we have investigated measures of intra-gene methylation architecture, and their ability to differentiate between healthy and disease phenotypes. For this we have developed new measures, and adapted standard ones.

## Results

**Figure 1:**
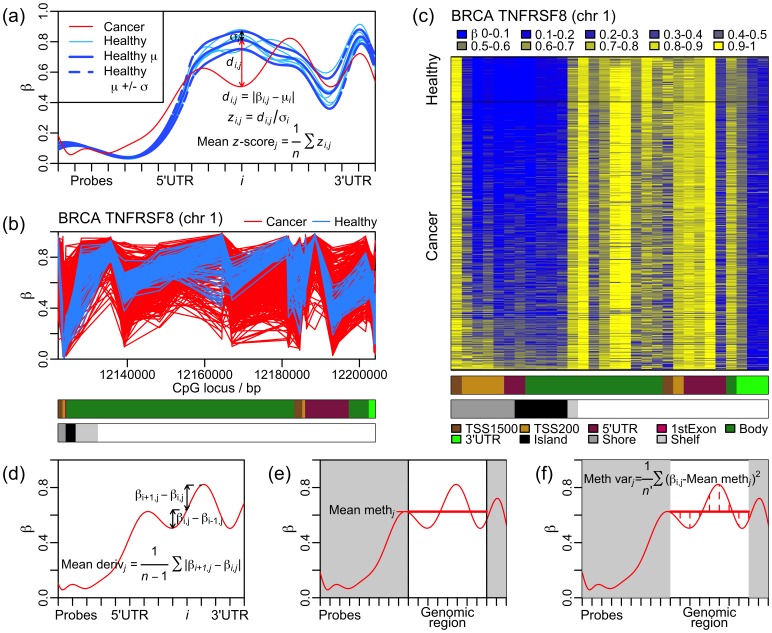
Per-gene methylation measures. (a) The mean 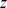-score measure is calculated for tumour sample 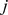 (shown in red) for gene 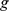 (to which 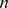 probes map), from the mean, 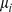, and standard deviation, 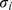, of the healthy control samples at each probe 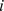 (b) The methylation profiles of 586 cancer (red) and 98 healthy (blue) samples across a gene found as significant according to the mean 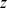-score measure, with probes spaced (unevenly) according to their genomic loci. Genomic regions are indicated under the gene with the colour code displayed at the bottom of the figure. (c) A heatmap illustrating the same gene, with probes evenly spaced; 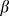 values for each sample and each probe are indicated by the colour code displayed at the top of the figure. Samples are plotted in order of mean 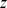-score, such that the tumour sample with the smallest mean 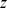-score and the healthy sample with the smallest mean 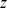-score are adjacent. Genomic regions are indicated under the gene with the colour code displayed at the bottom of the figure. N.B., this gene has two transcriptional start sites (TSSs) in different locations. (d) The mean derivative measure is calculated, for sample 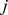, as the mean of the absolute differences in the corresponding 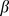 values between consecutive probes, across the whole of gene 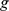. (e) The mean methylation measure is calculated, for sample 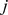, as the mean of the corresponding 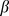 values of the probes annotated to a particular genomic region of 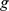. (f) The methylation variance measure is calculated, for sample 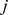, as the variance of the corresponding 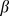 values of the 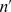 probes annotated to a particular genomic region of 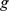. N.B., (d)-(f) are calculated without reference to healthy samples, whereas (a) is calculated with reference to healthy samples.

To investigate intra-gene methylation architecture, four gene-centric measures are considered, as follows:

1. The mean deviation of the sample methylation profile from the mean methylation profile of healthy phenotype control samples, for each gene. This mean methylation profile may fluctuate a lot within each gene, and so it is not the same as the mean methylation level of a gene. Because this mean deviation is normalised at every probe by dividing by the probe standard deviation across the healthy phenotype control samples, it is called the ‘mean 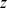-score’ measure; this is illustrated in Figure 1(a). An example of one of the genes found to be most significant according to this measure is shown in Figure 1(b) and (c).
2. The mean derivative of the methylation measurements for each gene. The derivative of the methylation profile for a given gene and sample is approximated by the differences between the methylation values measured at consecutive probes mapping to that gene. The mean of the absolute values of these differences is then calculated as the ‘mean derivative’ measure; this is the same as the sum total of all the increases and decreases in methylation level from one probe to the next across the gene; this is illustrated in Figure 1(d). This is a self-calibrating measure of intra-gene methylation variability, because it is calculated for a given sample from the differences within that sample, and without reference to any other sample.
3. The mean of the methylation measurements for a particular genomic region for each gene; this is illustrated in Figure 1(e). Typical mean methylation levels vary greatly from one genomic region to another; hence the mean methylation level for a particular genomic region was used as the ‘mean methylation measure’ for a gene, and the same region was used for each gene.
4. The variance for each gene of the methylation measurements for a particular genomic region; this is illustrated in Figure 1(f). Because variance is calculated in relation to the mean, this measure was similarly calculated for each gene using only the probes mapping to a particular genomic region, again using the same genomic region for each gene. This is called the ‘methylation variance’ measure; it is another self-calibrating measure.

These four measures each seek to examine a different characteristic of intra-gene methylation architecture, and all are able to classify samples one-by-one, i.e., they are intra-gene or intra-sample measures, rather than sample to sample measures as has been investigated previously in the context of methylation variability.

As the mean 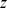-score is calculated as a mean measure of methylation difference from the healthy methylation profile, strictly speaking it is a measure of methylation instability. The mean derivative and methylation variance measures are both measures of intra-gene methylation variability; however, the mean derivative is calculated with reference to the ordering of the probes (i.e., this measure would return a different number if the order of the probes was randomised) whereas the methylation variance would not; the mean derivative additionally considers all probes mapping to the gene, whereas the methylation variance measure only considers probes mapping to a particular genomic region. The mean methylation measure is unique here in that it does not measure difference in methylation level and instead measures absolute methylation level; it is included here mainly for comparison.

The properties of these four measures were initially investigated in the context of fourteen Illumina Infinium Human Methylation 450 data sets, which were downloaded from The Cancer Genome Atlas (TCGA) [19]. We applied these four measures to the fourteen TCGA data sets; in all, we analysed 450 K DNAm data from 3284 tumour and 681 healthy samples; details of the number of samples of each phenotype and in each data set are shown in Table 1 (for data set abbreviations, see ‘Methods and Models’). We also carried out a meta-analysis of these data which is to our knowledge the largest meta-analysis performed in any DNA methylation study.

**Table 1:** Number of samples in each data set.

	healthy	cancer	total
BRCA	98	586	684
UCEC	36	334	370
THCA	50	357	407
LUAD	32	306	338
BLCA	18	126	144
LUSC	43	227	270
COAD	38	258	296
HNSC	50	310	360
KIRC	160	283	443
LIHC	50	98	148
READ	7	96	103
PRAD	49	176	225
KIRP	44	87	131
PAAD	6	40	46

### Comparison of Intra-gene Methylation Measures

**Figure 2:**
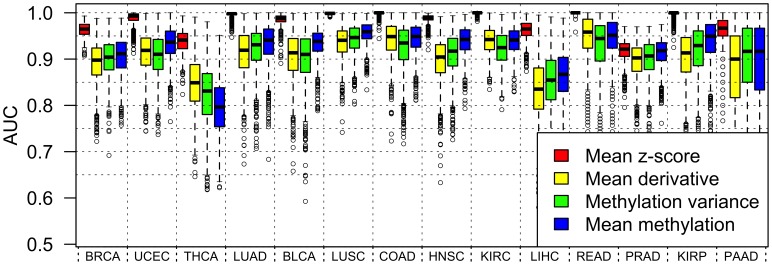
Distributions of per-gene AUCs calculated from intra-gene methylation measures. Each box displays the values of the AUCs for the 1000 most significant genes for a particular tumour type and intra-gene methylation measure. The mean 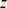-score predicts phenotype better than the other three measures in all 14 tumour types. Tumour type abbreviations are as follows: Bladder Urothelial Carcinoma (BLCA), Breast Invasive Carcinoma (BRCA), Colon Adenocarcinoma (COAD), Head and Neck Squamous Cell Carcinoma (HNSC), Kidney Renal Clear Cell Carcinoma (KIRC), Kidney Renal Papillary Cell Carcinoma (KIRP), Liver (LIHC), Lung Adenocarcinoma (LUAD), Lung Squamous Cell Carcinoma (LUSC), Pancreatic Adenocarcinoma (PAAD), Prostate Adenocarcinoma (PRAD), Rectum Adenocarcinoma (READ), Thyroid Carcinoma (THCA), and Uterine Corpus Endometrioid Carcinoma (UCEC).

As a preliminary assessment of the relative merits of these four measures, we looked at their ability to distinguish between tumour and healthy tissue. The correlation of the tissue sample phenotype to the four methylation measures was considered in terms of distributions of per-gene AUCs (area under curve, which is a measure of prediction accuracy, see ‘Methods and Models’ for details). These distributions are shown in box-plots in Figure 2. For every data set, the mean 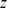-score measure is significantly better at discriminating tumour from healthy tissue using these methylation data, than the mean derivative measure, the methylation variance measure, and the mean methylation measure (visual comparison of Figure 2 was confirmed by Kolmogorov-Smirnov tests, data not shown); this is because the mean 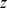-score measure is defined relative to the healthy mean methylation profile. Excluding the mean 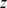-score measure, the mean methylation measure is significantly better at discriminating tumour from healthy tissue than the remaining two measures in ten of the remaining data sets, with the mean derivative discriminating significantly better in two data sets (READ and THCA), and inconclusive results for the remaining data sets (KIRC and PAAD, which has unstable results due to small sample size). Figure S3 shows, in scatter plots, pairwise comparisons of each of the four methylation measures for a gene which was among the top 1000 genes with the highest AUC according to each of these measures.

To directly compare the effectiveness of the mean 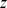-score measure at predicting phenotype (cancer/healthy) independent of mean methylation level, a logistic regression model was fitted to each gene using mean 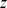-score and mean methylation as covariates, leading to 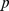-values for each gene for each of mean 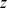-score and mean methylation. In every data set except two, for the large majority (80–100%) of those genes with at least one of the two covariates significant, the mean 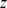-score covariate 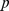-value was more significant than the corresponding mean methylation covariate 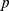-value. In the remaining two data sets, the mean 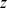-score covariate 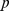-value was more significant for the majority (50–80%) of genes with at least one significant covariate (detailed results not shown). Hence, the mean 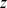-score is a better predictor of phenotype than the mean methylation, even after adjustment for mean methylation level.

### Meta-analysis and Gene-set Enrichment Analysis

**Figure 3:**
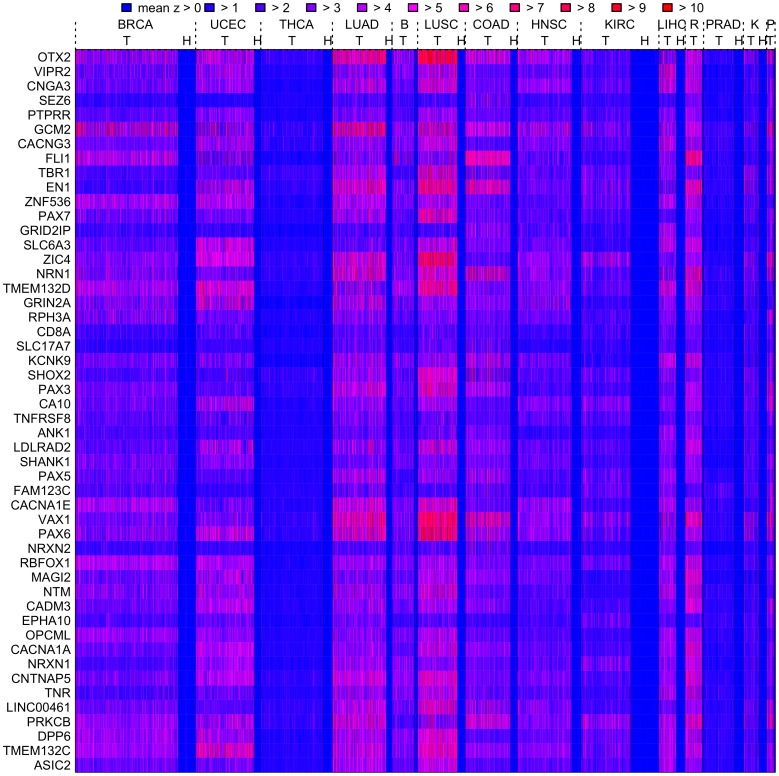
Heatmap of the mean 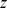-score for the top 50 genes found by the meta-analysis. Mean 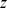-scores for tumour (T) and healthy (H) samples are displayed in a heatmap according to the colour code for the top 50 meta-analysis genes (top 50 most consistently unstable genes). The heatmap shows the extent to which the instability is consistent (high mean 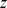-score, red) across cancer patients as compared to healthy patients (low mean 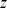-score, blue). For each tissue type healthy samples appear to the right of tumour samples; where no space is available the (H) label is omitted. Abbreviations: R (READ), B (BLCA), K(KIRP), P (PAAD).

A meta-analysis of the fourteen data sets was carried out. Genes were assigned significance according to their mean AUC (based on the mean 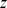-score measure) across all data sets by a permutation method (see ‘Methods and Models’ for details); this identified over 4000 significant genes which were associated with a consistent difference between cancer and healthy phenotypes across tissue types (FDR 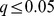). These genes consistently show the biggest differences between healthy and cancer phenotypes (as the mean 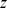-score measure is defined relative to healthy control samples), and as the mean 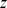-score is a measure of methylation instability, they are termed the most unstable meta-analysis genes. The mean 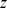-scores for individual tumour and healthy samples for the 50 most significant of these most unstable meta-analysis genes are displayed in Figure 3, and details about the 100 most significant of these genes are shown in Table S1. In particular, Figure 3 shows the extent to which the instability is consistent (high mean 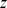-score, red) across cancer patients as compared to healthy patients (low mean 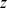-score, blue). Genes with a mean AUC close to 0.5 across most tumour types were also found; these are genes which tend to have the smallest differences between healthy and cancer phenotypes across tissue types and hence are marked as least unstable meta-analysis genes. Over 2800 least unstable meta-analysis genes were found to be significant by this permutation method (FDR 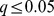) and the 100 most significant of these are shown in Table S2. There is however less consistency among the least unstable meta-analysis genes across tumour types, e.g., the 100th placed significant least unstable meta-analysis gene has an AUC of less than 0.6 for only 10 out of 14 tumour types.

To confirm the biological significance of the findings of this meta-analysis with reference to genes which are well known to be important in cancer biology, the most unstable and least unstable meta-analysis genes were tested for enrichment by genes which in ES cells carry the repressing/activating chromatin marks H3K27me3 (H3K27 ES genes), H3K4me3 (H3K4 ES genes) and bivalent (i.e., both H3K27me3 and H3K4me3 marks, Biv ES genes) and enrichment by PCGTs (ES cell polycomb group targets). The most unstable meta-analysis genes are highly enriched by Biv and H3K27 ES genes and PCGTs, and the least unstable meta-analysis genes are highly enriched by H3K4 ES genes (Table 2).

**Table 2:** Enrichment of MUs and LUs genes by stem cell genes.

	H3K27	H3K4	Biv	PCGT
MUs	1.43×10^–28^	1	5.19×10^–278^	1.77×10^–234^
LUs	1	4.33×10^–70^	1	1

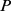
-values (one-sided Fisher’s exact test) show enrichment of most unstable meta-analysis genes (MUs) and enrichment of least unstable meta-analysis genes (LUs) by genes in various SC categories. This confirms the biological significance of the findings of the meta-analysis with reference to these genes which are well known to be important in cancer biology.

A more general gene-set enrichment analysis (GSEA) was also carried out, testing enrichment of the most unstable and least unstable meta-analysis genes by members of over 6000 gene sets (see ‘Methods and Models’ section for details). The 100 most significantly enriched of these gene sets by the most unstable and least unstable meta-analysis genes appear in tables S3 and S4 respectively. In particular Table S3 (gene sets enriched by most unstable meta-analysis genes) shows many developmental and cell signalling gene sets.

The most unstable meta-analysis genes are associated with generally higher methylation levels than genes which are not significant according to the meta-analysis (i.e., genes which are neither most unstable or least unstable meta-analysis genes) for both tumour and healthy samples, for these genomic regions located closer to the promoter across all tissue types, however the most unstable meta-analysis genes are also associated with a large variability of methylation levels (Figure S4). The least unstable meta-analysis genes conversely are associated with consistently very low levels of methylation in both tumour and healthy samples for these genomic regions, and particularly for TSS200, 5′UTR and 1stExon, suggesting that the low methylation instability of these genes is associated with a lack of methylation in the most functionally important genomic regions in both diseased and normal tissues, and therefore that regulation of these genes is by mechanisms other than those involving DNA methylation, in particular the availability of transcription factors.

### Correlation of Tumour Gene Expression with Intra-gene Methylation Architecture

**Figure 4:**
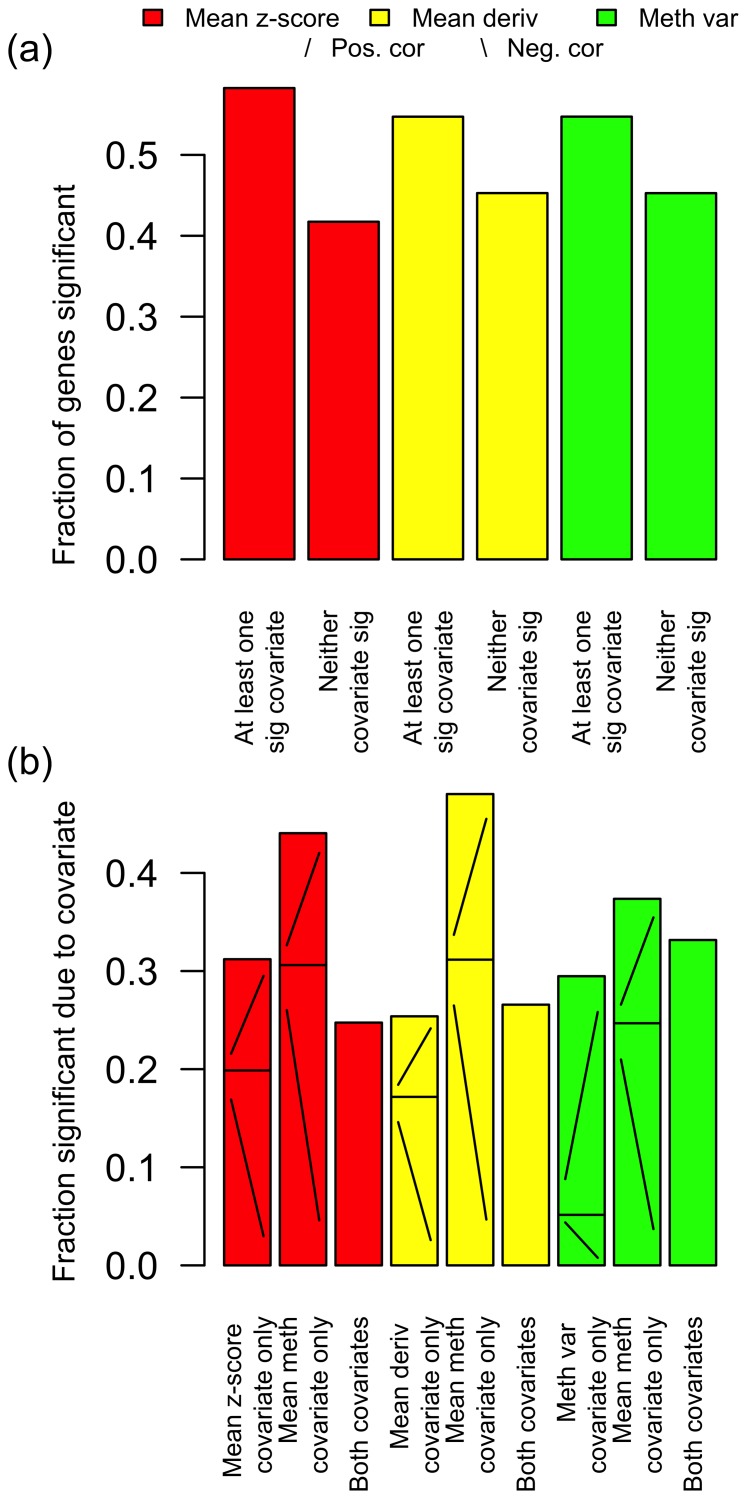
Correlation of expression to intra-gene methylation architecture, for matched BRCA samples. Expression was taken as the response variable, with one of mean 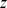-score, mean derivative and methylation variance as one covariate predictor, together with mean methylation as a second covariate predictor. (a) The proportion of genes with at least one covariate significant (FDR 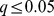), and the proportion of genes with neither covariate significant. (b) The proportion of significant genes (i.e., the proportion of the genes represented by the left of each pair of bars in a) which are significant due to one, or the other, or both covariates. For the genes which are significant due to only one covariate predictor, the proportions of these genes for which the significance is due to positive or negative correlation are indicated on the bars with/and \ respectively. There are many genes with expression not significantly predicted by mean methylation but significantly predicted by mean 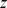-score, mean derivative, or methylation variance.

In order to investigate the effect of intra-gene methylation architecture on gene expression, the 217 BRCA tumour samples with matched gene expression and methylation data available from TCGA were considered in more detail. For each gene a non-linear multivariate regression analysis was performed (see ‘Methods and Models’) of gene expression to intra-gene methylation architecture, for these matched tumour samples, taking gene expression as the response, and taking one of mean 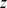-score, mean derivative and methylation variance as one covariate predictor, together with mean methylation as a second covariate predictor. The relative proportions of genes found as significant or not, and significant according to one covariate or the other, or both, are shown in Figure 4; in particular there are many genes with expression not significantly predicted by mean methylation but significantly predicted by mean 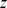-score, mean derivative, or methylation variance.

Enrichment by stem cell genes of genes with expression significantly predicted by only one covariate was again tested to confirm the biological significance of findings with reference to genes which are well known to be important in cancer biology. It was found that genes with expression predicted by only the mean 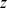-score covariate were significantly enriched by Biv ES genes and PCGTs (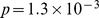 and 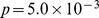 respectively, Fisher’s exact test), a result which is consistent with the findings here that Biv ES genes are enriched among the most unstable meta-analysis genes, i.e., those genes which are most consistently associated with the biggest difference in methylation pattern between cancer and healthy phenotypes. It was also found that, correspondingly, genes with expression predicted by only the mean methylation covariate in the multivariate regression with the mean 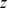-score covariate were significantly enriched (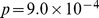, Fisher’s exact test) by H3K4 ES genes, a result which is consistent with our findings that H3K4 ES genes are enriched among least unstable meta-analysis genes, i.e., those genes which have consistently least difference in methylation pattern between cancer and healthy phenotypes. Similarly, it was found that genes with expression predicted by only the mean derivative covariate were significantly enriched by Biv ES genes and PCGTs (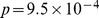 and 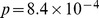 respectively, Fisher’s exact test) and that genes with expression predicted only by the mean methylation covariate in the same multivariate regression were significantly enriched by H3K4 ES genes (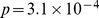, Fisher’s exact test).

These findings extend to heterogeneous tumour phenotype, as defined by gene expression, the idea that differences in methylation patterns in stem cell genes are a hallmark of cancer, and shows that this can be measured by intra-gene methylation architecture in the form of intra-gene methylation variability (according to the mean derivative and methylation variance measures) and instability (according to the mean 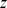-score measure) more accurately than by mean methylation level alone.

### Association of Genome-wide Mean 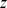-score with Breast Cancer Intrinsic Subtypes

**Figure 5:**
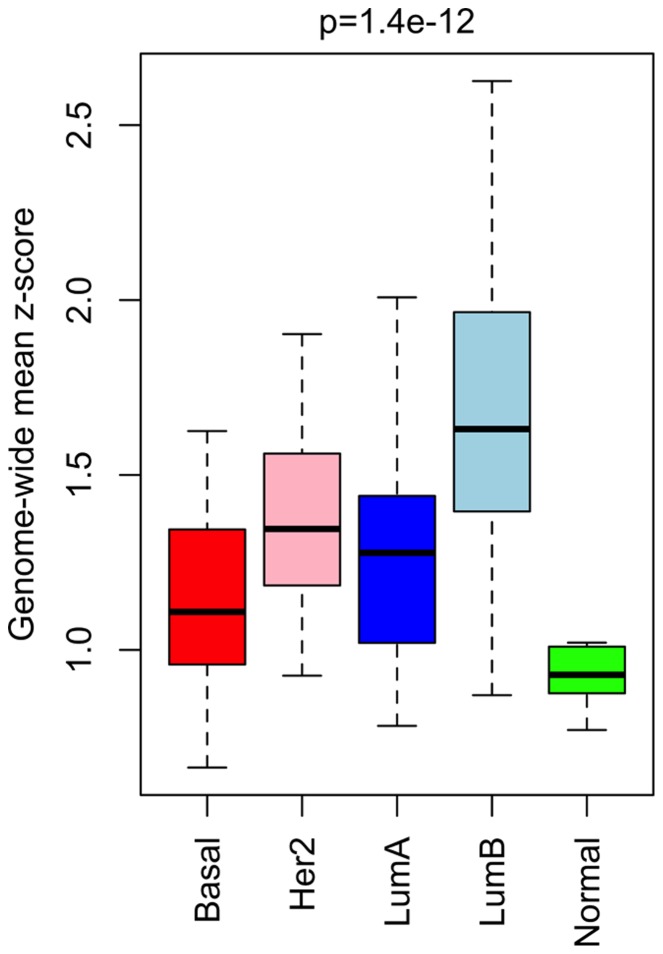
Distributions of genome-wide mean 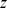-score, for breast cancer intrinsic subtypes. The mean across all genes of the mean 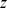-scores was calculated for the 217 BRCA samples with matched expression and methylation data available. These samples were independently classified by correlation of their gene expression profiles (Spearman correlation) with those of the PAM50 breast cancer intrinsic subtype classifier [20]. The distributions of these genome-wide mean 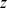-scores, for each intrinsic subtype, are shown in the boxplots. Indicated significance was calculated using the Kruskal-Wallis test.

Differences in intra-gene methylation architecture between heterogenous tumour phenotypes (as defined by gene expression) was further explored, in the context of breast cancer intrinsic subtypes. The same 217 BRCA samples with matched gene expression and methylation data available were each uniquely assigned to one of these disease subtypes, according to established molecular definitions, using the PAM50 classifier [20]. This was done by correlating the gene expression profile (Spearman correlation) for each sample to the PAM50 classifier canonical gene expression profiles for 5 different intrinsic subtypes, and for each sample choosing the subtype with the largest correlation coefficient, leading to 42 samples classified as Basal, 24 as Her2, 81 Luminal A, 54 Luminal B, and 16 classified as Normal. For each of these samples, a genome-wide mean 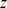-score was also calculated, as a per-sample genome-wide measure of intra-gene methylation architecture. The distributions of these genome-wide mean 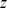-scores for each intrinsic subtype are shown in Figure 5; there are clear differences in the means and distributions between each of the subtypes. A Kruskal-Wallis test was carried out to check the significance of these differences, with a very significant result, 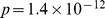. Removing the samples classified as Luminal B and Normal (as the distributions of genome-wide mean-z scores have larger and smaller variances, respectively, for these subtypes than the others), still resulted in a significant result in the Kruskal-Wallis test, 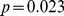. This ability to distinguish between heterogenous tumour phenotypes, in the context of established molecular definitions of disease subtypes, indicates that it may be possible to use intra-gene methylation architecture to develop new molecular classifiers of cancer, or make established ones more robust. This is particularly interesting, since methylation levels are typically more stable than gene expression levels.

## Discussion

We have shown that the reorganisation of intra-gene methylation architecture is a fundamental characteristic of cancer cells, and that there are many ways to assess these differences, which can provide complimentary information. We have developed measures to detect some of these differences, including the first investigation of intra-gene variability of methylation (as opposed to sample to sample variability of methylation). We have shown that our mean 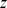-score measure is consistently more effective at predicting cancer compared to healthy phenotype than mean methylation, even after adjustment for the mean methylation level.

We have carried out what is, to our knowledge, the largest meta-analysis performed in any DNA methylation study. In particular, over 4000 genes were found to be significantly associated with a consistent difference between cancer and healthy phenotypes, demonstrating that, as a method for distinguishing cancer from healthy tissue, our mean 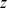-score measure is robust to differences between tumour types. The 100 most significant genes according to this meta-analysis (Table S1) can be considered as particularly characteristic of a generalised and non tissue-specific cancer phenotype. These least unstable meta-analysis genes are also significantly enriched (Table 2) by genes carrying H3K27 and bivalent chromatin marks in ES cells and by PCGTs, consistent with the idea that the tumour phenotype is associated with the acquisition of stem-like cell characteristics [15]. In this meta-analysis, over 2800 genes were also found to be significantly associated with an absence of difference in methylation pattern from healthy to cancer, and these are significantly enriched by genes carrying the activating H3K4 chromatin mark in ES cells (Table 2).

There is a particularly big contrast in the effectiveness of these methods with respect to endocrine cancers. On the one hand, these methods are particularly insensitive to PRAD and THCA (Figure 2), and furthermore, the genes identified as being most significant in the meta-analysis do not seem to show the same pattern of instability in these cancers (Figure 3). This suggests the possibility that epigenetic mechanisms may, in general, be less relevant to oncogenic processes in THCA and PRAD. On the other hand, These methods are very effective at determining differences in epigenetic patterns with respect to both BRCA and UCEC (Figure 2), and these cancers show significant patterns of instability in a large proportion of the genes identified as being most significant in the meta-analysis (Figure 3).

The correlation for tumour samples of gene expression to intra-gene methylation architecture (Figure 4) shows that there are a substantial number of genes for which mean methylation is not significantly predictive of gene expression but other measures of intra-gene methylation architecture are. In particular, in the case of our mean 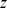-score and mean derivative measures, genes with expression predicted by these measures and not by mean methylation are enriched by Biv ES genes and PCGTs, suggesting that the intra-gene methylation instability and variability are able to provide important information about heterogeneous tumour phenotype (as measured by gene expression), particularly in relation to stem-like cell characteristics, which is beyond the reach of measures based on mean methylation level alone.

The differences in the genome-wide mean 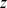-scores across breast cancer intrinsic subtypes (Figure 5) highlight the potential of intra-gene methylation architecture to distinguish between heterogenous tumour phenotypes in the context of established gene expression based definitions of distinct subtypes of this disease. This indicates that it may be possible to use intra-gene methylation architecture to develop new molecular classifiers of cancer, or make established ones more robust.

Further improvements in classification by our methods will be gained by the inclusion of complementary epigenetic data, in particular those which measure patterns of histone modification. As discussed, it is well established how crucial genes which carry important histone markings in stem cells are to understanding cancer biology. By extending the view of the epigenetic landscape beyond DNA methylation to consider also histone markings not just in stem cells but also in mature healthy cells and cancer cells, we will gain mechanistic insights into the interaction between intra-gene methylation architecture and histone modifications.

In summary, we have shown for the first time that generalised differences in intra-gene methylation architecture are a better predictor of phenotype than mean methylation level alone, and we have developed novel measures of these differences, which offer a considerable reduction in complexity from per CpG methylation measures (hundreds of thousands of features) to per gene methylation measures (tens of thousands of features). We have shown that there are many genes with expression predicted by measures of intra-gene methylation architecture other than mean methylation level, and therefore that more general measures of intra-gene methylation architecture offer novel information about heterogeneous tumour phenotype (as defined by gene expression). We have also shown that intra-gene methylation architecture is able to distinguish between established molecular definitions of heterogenous cancer subtypes. Because it has been shown previously that differences in methylation pattern occur prior to the onset of disease [18], we anticipate that our measures of intra-gene methylation architecture might also be able to efficiently find pre-disease methylation patterns. We therefore believe that our measures of intra-gene methylation architecture have potential for further development as DNA based cancer biomarkers.

## Methods and Models

### Data Source and Preprocessing

Methylation data, collected via the Illumina Infinium HumanMethylation450 platform, were downloaded from The Cancer Genome Atlas (TCGA) project [19] at level 3. These data were obtained from fourteen different tumour types, as follows: Bladder Urothelial Carcinoma (BLCA), Breast Invasive Carcinoma (BRCA), Colon Adenocarcinoma (COAD), Head and Neck Squamous Cell Carcinoma (HNSC), Kidney Renal Clear Cell Carcinoma (KIRC), Kidney Renal Papillary Cell Carcinoma (KIRP), Liver (LIHC), Lung Adenocarcinoma (LUAD), Lung Squamous Cell Carcinoma (LUSC), Pancreatic Adenocarcinoma (PAAD), Prostate Adenocarcinoma (PRAD), Rectum Adenocarcinoma (READ), Thyroid Carcinoma (THCA), and Uterine Corpus Endometrioid Carcinoma (UCEC).

These data were pre-processed by first removing probes with non-unique mappings and which map to SNPs (as identified in the TCGA level 3 data); probes mapping to sex chromosomes were also removed; in total 98384 probes were removed in this way from all data sets. After removal of these probes, 270985 probes with known gene annotations remained. Individually for each data set, probes were then removed if they had less than 95% coverage across samples; probe values were also replaced if they had corresponding detection 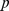-value greater than 5%, by KNN (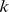 nearest neighbour) imputation (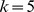).

Matched gene expression data were also downloaded for 217 samples for the BRCA data set, and were quantile normalised.

### Intra-gene Methylation Measures

Four methylation measures were considered, and were calculated separately for each sample, for each gene:

- ‘Mean 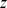-score’: the mean of the 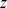-scores calculated from the methylation values for the probes mapping to the gene, with population parameters for each probe calculated from healthy control samples
- ‘Mean derivative’: the mean absolute derivative of the methylation profile across the gene
- ‘Methylation variance’: the variance of the methylation values for probes mapping to one genomic region of the gene
- ‘Mean methylation’: the mean of the methylation values for probes mapping to one genomic region of the gene

To calculate the mean of the 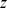-scores for each gene, the R [21]/Bioconductor [22] package ‘IlluminaHumanMethylation450k’ [23] was used to identify the probes mapping to each gene. Then for each probe, the mean and standard deviation of the methylation values for that probe were found from healthy tissue samples, allowing a 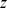-score 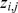 for each probe 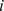, and for each sample 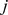, to be calculated according to equation 1. By taking the mean of the absolute 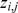 for all probes 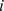 mapping to gene 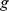, a single intra-gene methylation predictor value 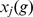 was then calculated for each gene 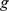, for each sample 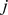, according to equation 2. A regularisation parameter, 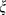, was added to each probe standard deviation when calculating probe 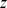-scores to prevent very large values from occurring; 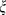 was chosen to be 0.01 after considering the distribution of probe standard deviations (Figure S5).
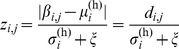
(1)

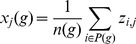
(2)where 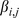 is the methylation value for probe 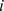 and sample 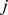, 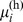 and 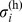 are the mean and standard deviation of the methylation values corresponding to the relevant healthy tissue samples for probe 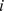, 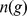 denotes the number of probes mapping to gene 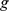 and 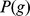 is the set of probes mapping to gene 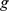.

To calculate the ‘mean derivative’ methylation measure, the ‘IlluminaHumanMethylation450k’ package was again used to find the probes mapping to each gene. Ordering the probes 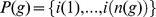 mapping to gene 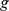 as they are positioned along the DNA, the derivative of the methylation profile for gene 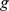 and sample 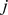 is estimated as the differences between the beta values at consecutive probes; hence the mean derivative for this gene and sample is estimated according to equation 3.
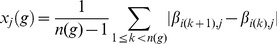
(3)


In this way, a single intra-gene methylation predictor value 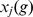 was calculated for each gene 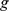, for each sample 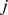.

To calculate the ‘methylation variance’ and ‘mean methylation’ measures, first the most effective genomic region, for each of these measures, across which to calculate these measures for each gene, was selected. For this, annotation information for the probes used by the Illumina Infinium platform was obtained from Gene Expression Omnibus (GEO) [24]. This annotation information details which probes map to one of six genomic regions for each gene, as follows: (1) TSS1500; probes annotated to distances greater than 200 bp and less than 1500 bp upstream from the TSS (transcriptional start site) of the gene. (2) TSS200; probes annotated to within 200 bp upstream of the TSS of the gene. (3) 5′UTR; probes annotated to the 5-prime untranslated region of the gene. (4) 1stExon; probes annotated to the first exon of the gene. (5) Body; other probes annotated to the gene body. (6) 3′UTR; probes annotated to the 3-prime untranslated region of the gene.

Separately for each of these genomic regions, the variance of methylation levels for each gene for probes mapping to the genomic region in question was calculated. Then the effectiveness of each genomic region at discriminating between healthy and tumour tissue was compared, by considering the correlation of the tissue sample phenotype to the methylation variance measure in terms of distributions of per-gene AUCs; the ‘Body’ (gene body) genomic region was chosen for the methylation variance measure, as it performed best in 13 out of 14 data sets (Figure S1). This methylation variance was calculated for each gene for which there was ‘Body’ annotation information available, to give a single intra-gene methylation predictor value 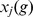, for each gene 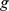, for each sample 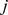.

It should be noted, however, that in general for each gene there were far more probes annotated as ‘Body’ than for other genomic regions (Table 3), leading to better estimates of the methylation variance for this region. Therefore, the relative greater effectiveness of this genomic region in this comparison does not necessarily imply biological significance. The minimum number of probes to be able to calculate the methylation variance for a given gene and genomic region was set to be 3, and the methylation variance was not calculated for any gene with any fewer annotated probes than this for a given genomic region. As there were more genes with at least 3 probes annotated to the ‘Body’ region (Table 3), it would be expected that there would be more genes which significantly associate with phenotype when this genomic region is used, which is likely to be another reason it performs better, without relevance to biological significance.

**Table 3:** Number of probes per genomic region and gene, of 18272 annotated genes.

	TSS1500	TSS200	5′UTR	1stExon	Body	3′UTR
Mean no.probes	2.7	2.4	2.5	1.5	7	0.82
Median no.probes	2	2	1	1	3	1
No. probes,95% CI	(0–10)	(0–7)	(0–13)	(0–6)	(0–39)	(0–4)
No. geneswith min3 probes	8512	7570	5258	3734	10029	958
No. geneswith min1 probe	14259	12979	11408	12194	15858	10291
No. geneswith 0probes	4013	5293	6864	6078	2414	7981

To choose which region to use to calculate the mean methylation measure, the same procedure was followed as for the methylation variance measure; the ‘Body’ genomic region was similarly chosen as this region correlated best with cancer/healthy phenotype in 10 out of 14 data sets (Figure S2). This mean methylation measure was calculated for each gene for which there was ‘Body’ annotation information available, to give a single intra-gene methylation predictor value 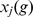, for each gene 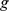, for each sample 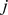. It is again worth noting that it is likely to be be due to the greater number of probes per gene annotated to ‘Body’, and the corresponding increase in accuracy of the calculated estimates of the mean methylation, which leads to this genomic region being more effective in this comparison, rather than there being any biological significance to this finding. In the case of mean methylation, it was only required that there be one probe annotated to a genomic region to allow a mean methylation level to be represented for that genomic region for that gene, as methylation levels of neighbouring CpGs within the same genomic region are expected to be highly correlated; again, there were more genes with at least one probe annotated to the ‘Body’ region than the other regions (Table 3), similarly suggesting a reason for its better performance other than biological significance.

### Comparison of Intra-gene Methylation Measures

Methylation measures were assessed according to the distributions of their per-gene AUCs. The AUC is the ROC (receiver-operator characteristic) ‘area under curve’ and is defined as the probability that a randomly chosen item from the ‘positive’ class will be scored higher than a randomly chosen item from the ‘negative’ class [25].

The same procedure was used for the main comparison of intra-gene methylation measures, for the choice of genomic region used in the methylation variance measure, and for the choice of genomic region used in the mean methylation measure. In this procedure, each data set was split half and half into a training and test set, maintaining the same proportion of cancer and healthy samples in both sets. Using only the training set, AUCs were calculated for all genes, and the top 1000 genes were selected as those with the best AUC. Then using the test set, an AUC was calculated for each of these top 1000 genes identified in the training set. For the mean 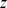-score measure, the mean healthy methylation profiles and healthy methylation standard deviations calculated from the training set were used to calculate the 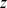-scores for both the cancer and healthy samples in the test set. The distributions of these test-set AUCs were compared in distribution density plots and using the Kolmogorov-Smirnov test (Figure 2 and Figures S1 and S2).

### Meta-analysis and Gene-set Enrichment Analysis

A meta-analysis of the fourteen data sets was carried out. The mean across all data sets of the per-gene AUCs generated from the mean 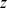-score measure was calculated for each gene. Significance was then assigned to each of these per-gene mean AUCs by similarly calculating null mean AUCs after permuting AUCs within data sets. This resulted in 4267 significant unstable meta-analysis genes with FDR 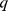-value [26] less than 5%, i.e., those genes corresponding to the upper tail of the null mean AUC distribution, which are associated with a consistent difference between cancer and healthy phenotypes across tissue types; the top 100 most significant of these unstable genes appear in Table S1. This permutation method also resulted in 2818 significant (FDR 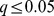) significant least unstable meta-analysis genes, i.e., those genes corresponding to the lower tail of the null mean AUC distribution, which were associated with least difference from healthy to cancer phenotype across tissue types the top 100 most significant of these least unstable genes appear in Table S2.

To confirm the biological significance of the findings of this meta-analysis with reference to genes which are well known to be important in cancer biology, the least unstable and least unstable meta-analysis genes were tested for enrichment by genes which in ES cells carry the repressing/activating chromatin marks H3K27me3 (H3K27 ES genes), H3K4me3 (H3K4 ES genes) and bivalent (i.e., both H3K27me3 and H3K4me3 marks, Biv ES genes) and enrichment by PCGTs (ES cell polycomb group target genes) using the one-tailed Fisher's exact test. A more general gene-set enrichment analysis (GSEA) was also carried out both on the least unstable and least unstable meta-analysis genes; 6811 gene set definitions were downloaded from the Broad Institute Molecular Signatures Database (http://www.broadinstitute.org/), and each gene set was tested separately for enrichment among the significant genes. Enrichment was again tested using the one-sided Fisher’s exact test, finding 1048 and 778 gene sets significantly (FDR 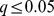) enriched by least unstable and least unstable meta-analysis genes respectively. The top 100 of these gene sets are shown in tables S3 and S4 respectively.

### Correlation of Tumour Gene Expression with Intra-gene Methylation Architecture

For the 217 BRCA tumour samples for which matched gene expression and methylation data were available, for each gene a multivariate regression analysis of gene expression and intra-gene methylation architecture was carried out. Gene expression was used as the response, with one of mean 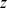-score, mean derivative and methylation variance as one covariate predictor, and with mean methylation as a second covariate predictor. As it was expected that this relationship would be non-linear, and as for a non-specified non-linear monotonic function the ranks of data points in response and predictor variables are linearly related if there is a good association between these variables, the ranks of each of the variables across the samples were correlated to one another, as follows.

Defining for gene 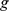 the ranks of the samples according to the expression data as 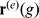, the ranks of the samples according to the mean 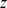-score, mean derivative or methylation variance as 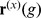, and the ranks of the samples according to the mean methylation as 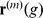, the data were modelled according to equation 4:
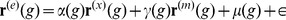
(4)where 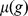 is the intercept term for gene 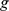, and  is the model error. Where 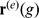 is well-correlated with 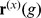, similar integer entries in these vectors (corresponding to similar ranks) will appear in similar positions in these vectors (N.B., these vectors are not themselves ordered). This will then be reflected as a small 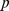-value for this comparison (calculated from the corresponding 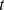-statistic for the linear model 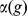 coefficient), and similarly for 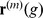 (and corresponding 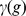 coefficient), if it is well-correlated with 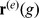.

This linear model was applied to the data for each gene present in the matched expression and methylation data for the BRCA dataset. ‘Body’ annotated probes were again used to calculate the methylation variance and mean methylation measures as used in this model, because probes annotated to this genomic region produced, in both cases, the greatest number of significant 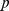-values (for the respective covariate), as compared to using probes annotated to each of the other genomic regions.

## Supporting Information

Figure S1 **Distributions of per-gene AUCs calculated from genomic feature methylation variance measures.** *P*-values shown are for Kolmogorov-Smirnov tests comparing the distributions of the most effective and second most effective measures.
(PDF)

Figure S2 **Distributions of per-gene AUCs calculated from genomic feature mean methylation measures.** *P*-values shown are for Kolmogorov-Smirnov tests comparing the distributions of the most effective and second most effective measures.
(PDF)

Figure S3 **Scatter plots showing pairwise comparisons of each of the four methylation measures, for the ONECUT3 gene.** ONECUT3 was among the top 1000 genes with the highest AUC according to each of the four methylation measures. There is one point in each scatter plot for each of the 98 healthy and 586 cancer samples in the BRCA data set.
(PDF)

Figure S4 **Genomic feature mean methylation levels for healthy and tumour samples.** (1) significant consistently most unstable genes in the meta-analysis (sig MUs) (2) genes not significant in the meta-analysis, (3) significant consistently least unstable genes in the meta-analysis (sig LUs).
(PDF)

Figure S5 **Distributions of probe standard deviations.** For each tumour type, the standard deviation of the beta values for each probe is found for cancer and for healthy samples; then estimates of the density distributions of the standard deviations for all probes are plotted for cancer and healthy samples for each tumour type. Locations of the modal standard deviation of each density distribution estimate are indicated with dashed lines, and are stated on each plot; where the distribution is multimodal the modal standard deviation corresponding to the greatest density is used.
(PDF)

Table S1 **Meta-analysis: 100 most significant most unstable genes.**
(PDF)

Table S2 **Meta-analysis: 100 most significant least unstable genes.**
(PDF)

Table S3 **Meta-analysis GSEA: most unstable genes, 100 most significant gene-sets.**
(PDF)

Table S4 **Meta-analysis GSEA: least unstable genes, 100 most significant gene-sets.**
(PDF)
