# Supplementary material for: Corruption of the Intra-Gene DNA Methylation Architecture Is a Hallmark of Cancer
Source: PLoS One. 2013 Jul 16;8(7):e68285. doi: 10.1371/journal.pone.0068285 (PMC3712966; doi:10.1371/journal.pone.0068285)
Supplement: Figure S1 — Distributions of per-gene AUCs calculated from genomic feature methylation variance measures. P-values shown are for Kolmogorov-Smirnov tests comparing the distributions of the most effective and second most effective measures. (PDF) [file pone.0068285.s001.pdf]

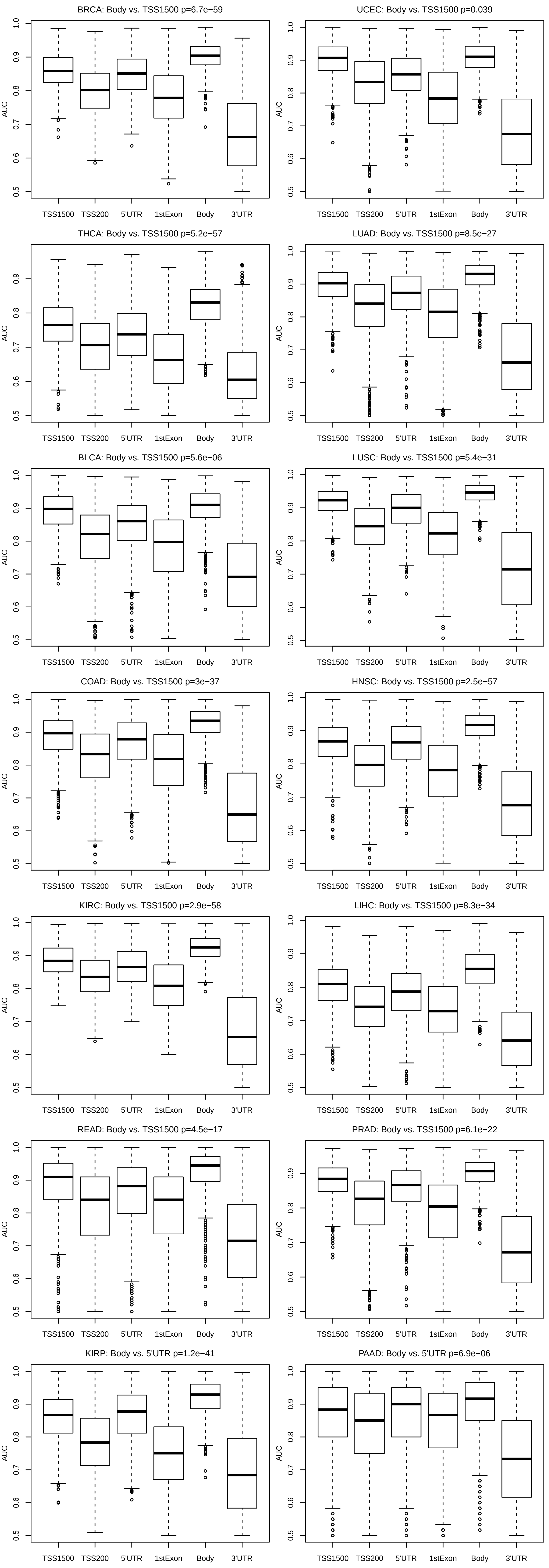

BRCA: Body vs. TSS1500 p=6.7e−59
UCEC: Body vs. TSS1500 p=0.039
THCA: Body vs. TSS1500 p=5.2e−57
LUAD: Body vs. TSS1500 p=8.5e−27
BLCA: Body vs. TSS1500 p=5.6e−06
LUSC: Body vs. TSS1500 p=5.4e−31
COAD: Body vs. TSS1500 p=3e−37
HNSC: Body vs. TSS1500 p=2.5e−57
KIRC: Body vs. TSS1500 p=2.9e−58
LIHC: Body vs. TSS1500 p=8.3e−34
READ: Body vs. TSS1500 p=4.5e−17
PRAD: Body vs. TSS1500 p=6.1e−22
KIRP: Body vs. 5'UTR p=1.2e−41
PAAD: Body vs. 5'UTR p=6.9e−06
AUC
0.5
0.6
0.7
0.8
0.9
1.0
TSS1500
TSS200
5'UTR
1stExon
Body
3'UTR
