# Supplementary material for: Corruption of the Intra-Gene DNA Methylation Architecture Is a Hallmark of Cancer
Source: PLoS One. 2013 Jul 16;8(7):e68285. doi: 10.1371/journal.pone.0068285 (PMC3712966; doi:10.1371/journal.pone.0068285)
Supplement: Figure S2 — Distributions of per-gene AUCs calculated from genomic feature mean methylation measures. P-values shown are for Kolmogorov-Smirnov tests comparing the distributions of the most effective and second most effective measures. (PDF) [file pone.0068285.s002.pdf]

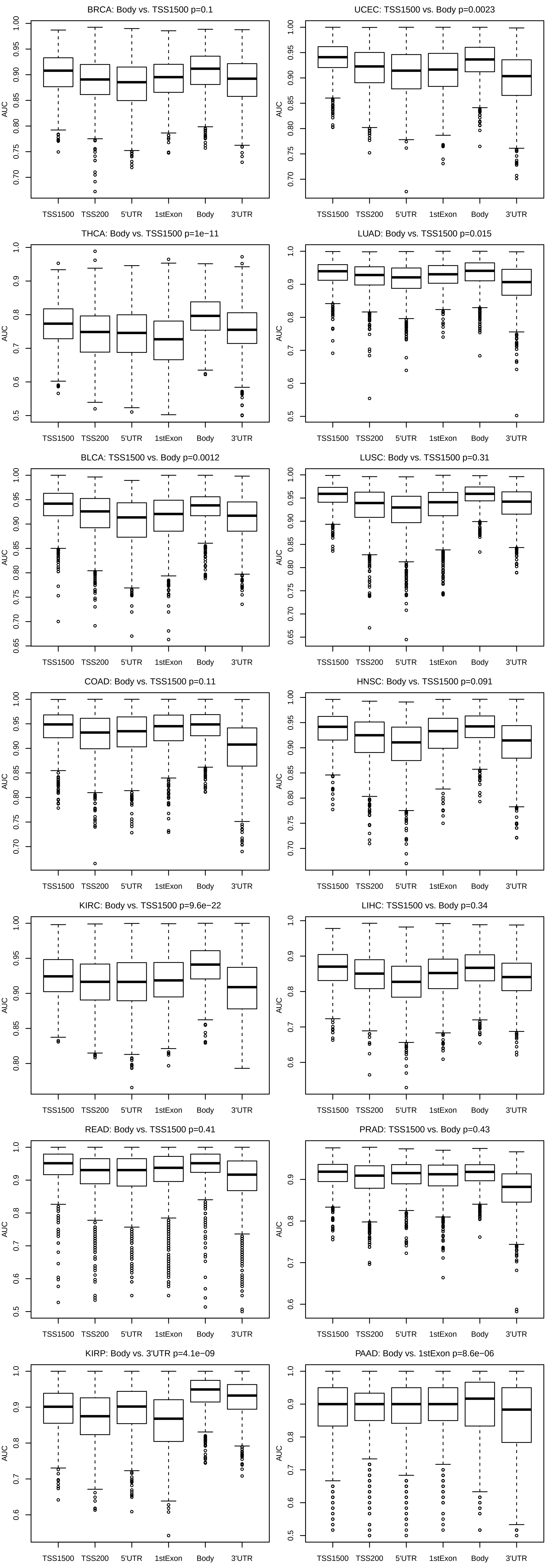
BRCA: Body vs. TSS1500 p=0.1
UCEC: TSS1500 vs. Body p=0.0023
THCA: Body vs. TSS1500 p=1e−11
LUAD: Body vs. TSS1500 p=0.015
BLCA: TSS1500 vs. Body p=0.0012
LUSC: Body vs. TSS1500 p=0.31
COAD: Body vs. TSS1500 p=0.11
HNSC: Body vs. TSS1500 p=0.091
KIRC: Body vs. TSS1500 p=9.6e−22
LIHC: TSS1500 vs. Body p=0.34
READ: Body vs. TSS1500 p=0.41
PRAD: TSS1500 vs. Body p=0.43
KIRP: Body vs. 3'UTR p=4.1e−09
PAAD: Body vs. 1stExon p=8.6e−06
AUC
TSS1500
TSS200
5'UTR
1stExon
Body
3'UTR
