# Supplementary figures and images for: Corruption of the Intra-Gene DNA Methylation Architecture Is a Hallmark of Cancer
Source: PLoS One. 2013 Jul 16;8(7):e68285. doi: 10.1371/journal.pone.0068285 (PMC3712966; doi:10.1371/journal.pone.0068285)

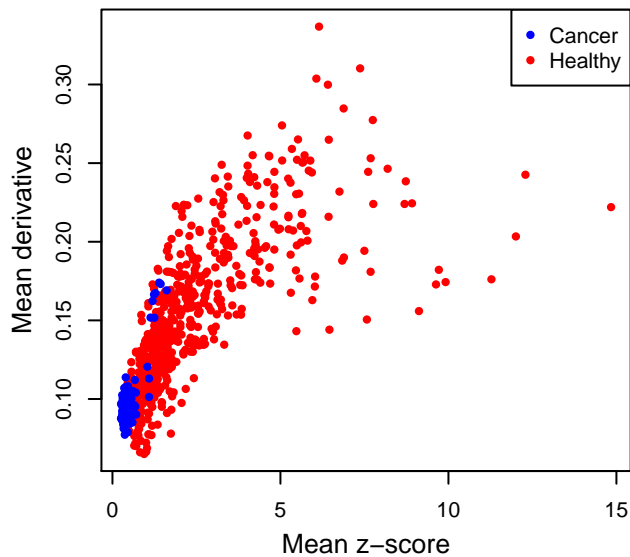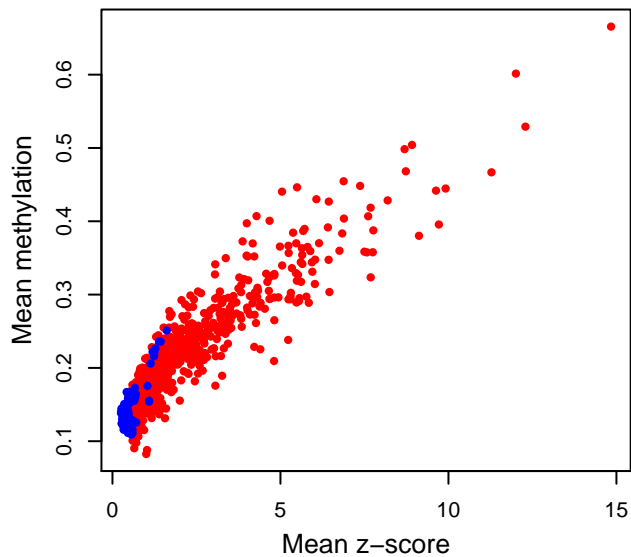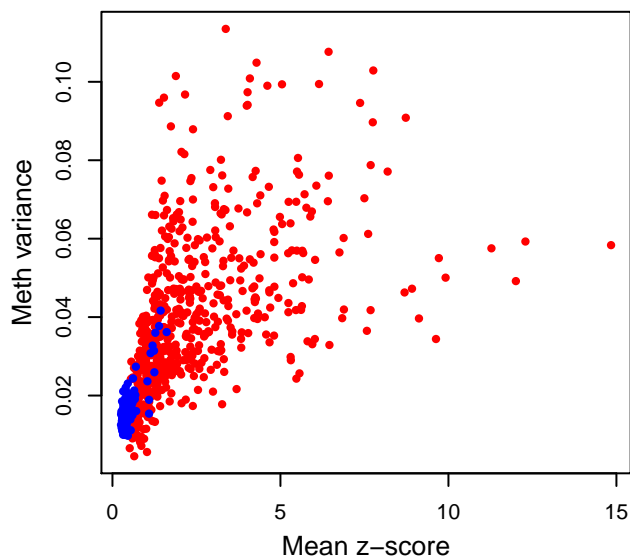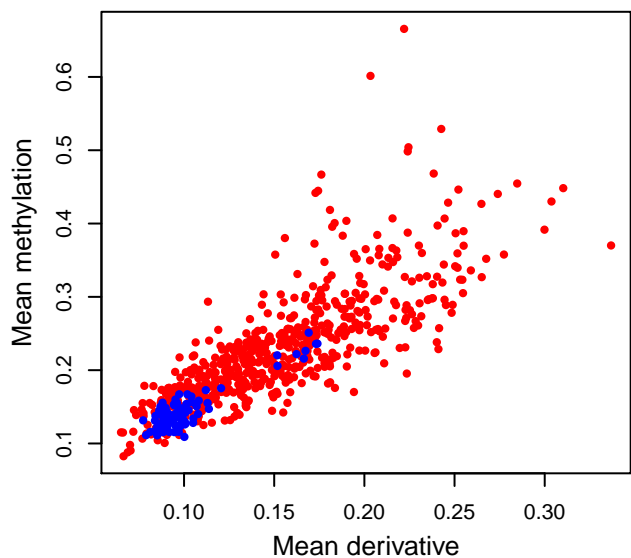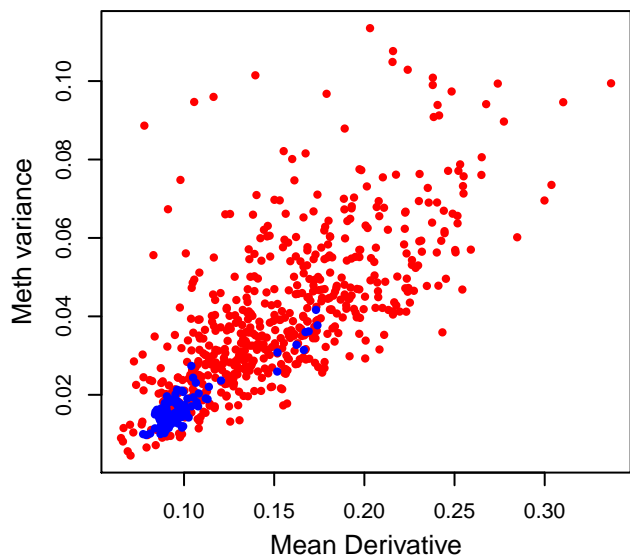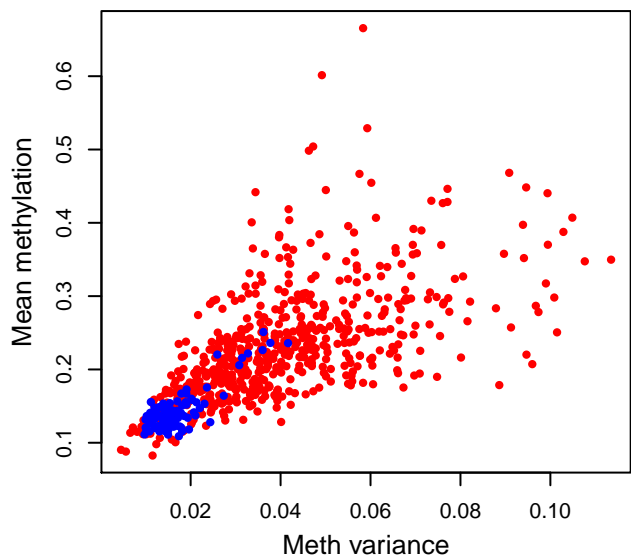

Supplement: Figure S3 — Scatter plots showing pairwise comparisons of each of the four methylation measures, for the ONECUT3 gene. ONECUT3 was among the top 1000 genes with the highest AUC according to each of the four methylation measures. There is one point in each scatter plot for each of the 98 healthy and 586 cancer samples in the BRCA data set. (PDF) [file pone.0068285.s003.pdf]

Mean methylation

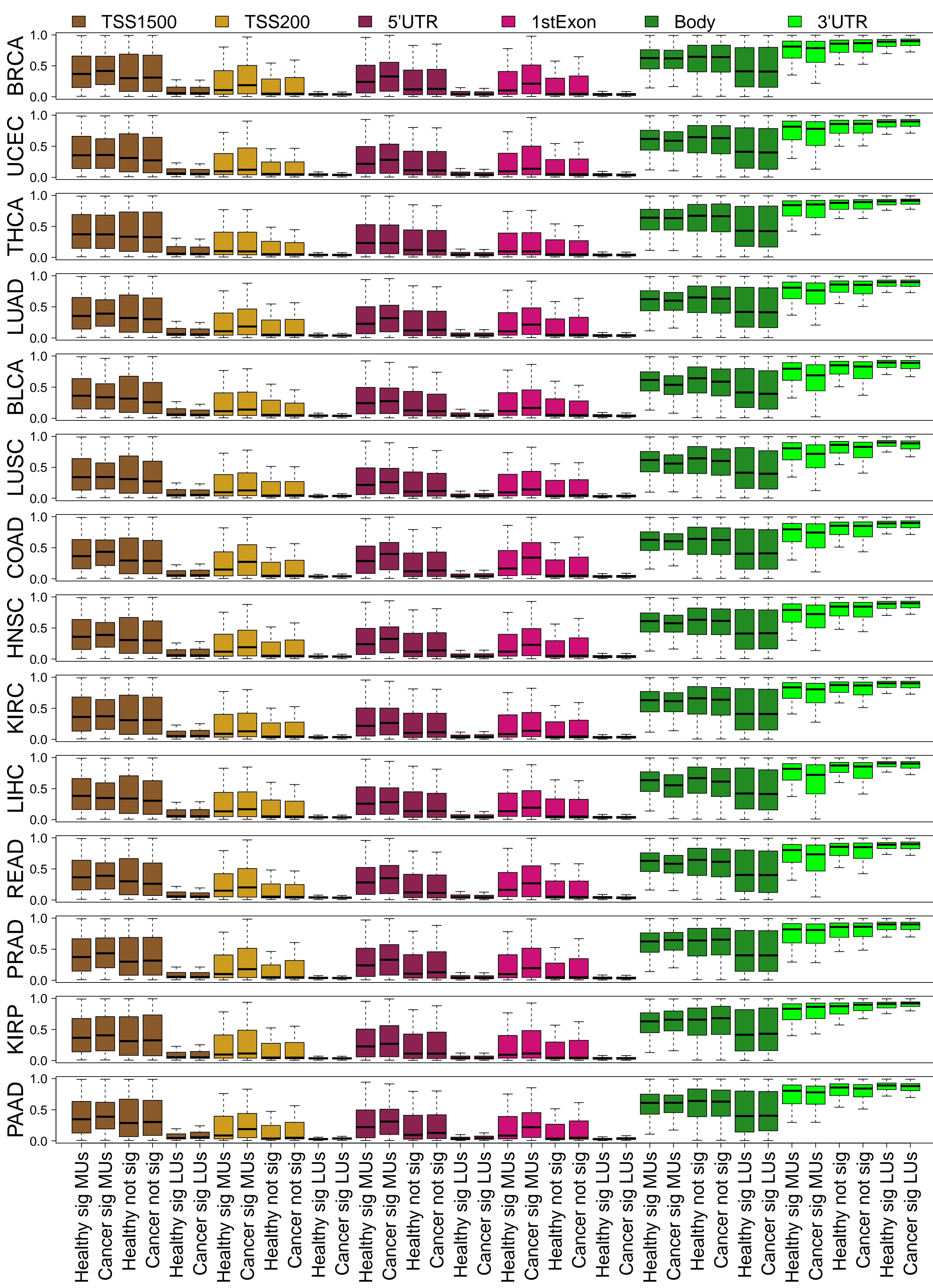

Supplement: Figure S4 — Genomic feature mean methylation levels for healthy and tumour samples. (1) significant consistently most unstable genes in the meta-analysis (sig MUs) (2) genes not significant in the meta-analysis, (3) significant consistently least unstable genes in the meta-analysis (sig LUs). (PDF) [file pone.0068285.s004.pdf]

— Cancer — Healthy

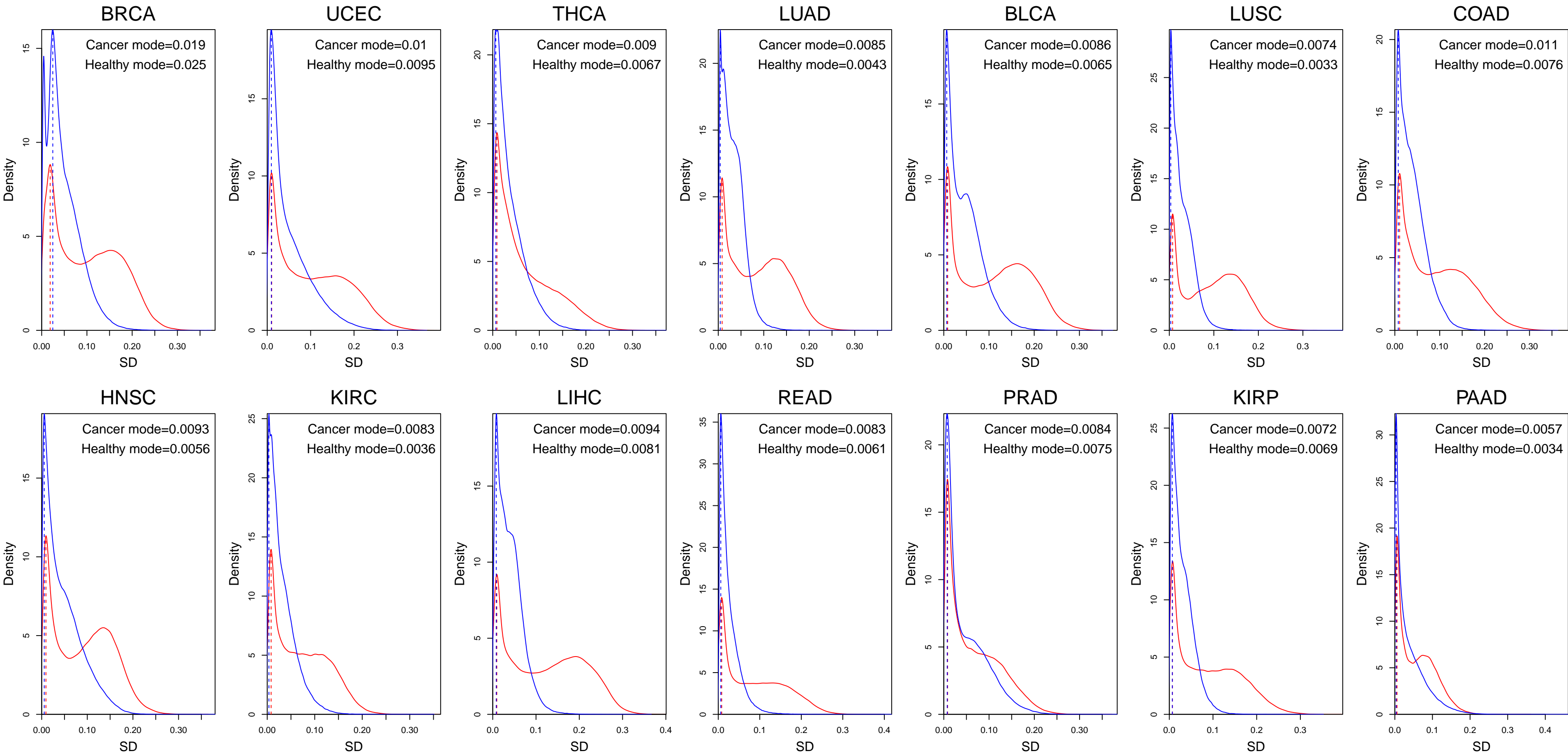

Supplement: Figure S5 — Distributions of probe standard deviations. For each tumour type, the standard deviation of the beta values for each probe is found for cancer and for healthy samples; then estimates of the density distributions of the standard deviations for all probes are plotted for cancer and healthy samples for each tumour type. Locations of the modal standard deviation of each density distribution estimate are indicated with dashed lines, and are stated on each plot; where the distribution is multimodal the modal standard deviation corresponding to the greatest density is used. (PDF) [file pone.0068285.s005.pdf]
