# Supplementary material for: Corruption of the Intra-Gene DNA Methylation Architecture Is a Hallmark of Cancer
Source: PLoS One. 2013 Jul 16;8(7):e68285. doi: 10.1371/journal.pone.0068285 (PMC3712966; doi:10.1371/journal.pone.0068285)
Supplement: Table S1 — Meta-analysis: 100 most significant most unstable genes. (PDF) [file pone.0068285.s006.pdf]

symbol	mean AUC	adj-p	Entrez	chr	band info	gene name
ASIC2	0.985	0	40	17	17q12	acid-sensing (proton-gated) ion channel 2
TMEM132C	0.985	0	92293	12	12q24.32	transmembrane protein 132C
DPP6	0.984	0	1804	7	7q36.2	dipeptidyl-peptidase 6
PRKCB	0.984	0	5579	16	16p11.2	protein kinase C, beta
LINC00461	0.983	0	645323	5	5q14.3	long intergenic non-protein coding RNA 461
TNR	0.983	0	7143	1	1q24	tenascin R (restrictin, janusin)
CNTNAP5	0.983	0	129684	2	2q14.3	contactin associated protein-like 5
NRXN1	0.983	0	9378	2	2p16.3	neurexin 1
CACNA1A	0.983	0	773	19	19p13	calcium channel, voltage-dependent, P/Q type, alpha 1A subunit
OPCML	0.982	0	4978	11	11q25	opioid binding protein/cell adhesion molecule-like
EPHA10	0.982	0	284656	1	1p34.3	EPH receptor A10
CADM3	0.982	0	57863	1	1q21.2-q22	cell adhesion molecule 3
NTM	0.982	0	50863	11	11q25	neurotrimin
MAGI2	0.982	0	9863	7	7q21	membrane associated guanylate kinase, WW and PDZ domain containing 2
RBFOX1	0.982	0	54715	16	16p13.3	RNA binding protein, fox-1 homolog (C. elegans) 1
NRXN2	0.981	0	9379	11	11q13	neurexin 2
PAX6	0.981	0	5080	11	11p13	paired box 6
VAX1	0.981	0	11023	10	10q26.1	ventral anterior homeobox 1
CACNA1E	0.981	0	777	1	1q25-q31	calcium channel, voltage-dependent, R type, alpha 1E subunit
FAM123C	0.981	0	205147	2	2q21.1	family with sequence similarity 123C
PAX5	0.981	0	5079	9	9p13	paired box 5
SHANK1	0.981	0	50944	19	19q13.3	SH3 and multiple ankyrin repeat domains 1
LDLRAD2	0.981	0	401944	1	1p36.12	low density lipoprotein receptor class A domain containing 2
ANK1	0.981	0	286	8	8p11.1	ankyrin 1, erythrocytic
TNFRSF8	0.981	0	943	1	1p36	tumor necrosis factor receptor superfamily, member 8
CA10	0.98	0	56934	17	17q21.33	carbonic anhydrase X
PAX3	0.98	0	5077	2	2q35	paired box 3
SHOX2	0.98	0	6474	3	3q25.32	short stature homeobox 2
KCNK9	0.98	0	51305	8	8q24.3	potassium channel, subfamily K, member 9
SLC17A7	0.98	0	57030	19	19q13	solute carrier family 17 (sodium-dependent inorganic phosphate cotransporter), member 7
CD8A	0.98	0	925	2	2p12	CD8a molecule
RPH3A	0.98	0	22895	12	12q24.13	rabphilin 3A homolog (mouse)
GRIN2A	0.98	0	2903	16	16p13.2	glutamate receptor, ionotropic, N-methyl D-aspartate 2A
TMEM132D	0.98	0	121256	12	12q24.33	transmembrane protein 132D
NRN1	0.98	0	51299	6	6p25.1	neuritin 1
ZIC4	0.98	0	84107	3	3q24	Zic family member 4
SLC6A3	0.98	0	6531	5	5p15.3	solute carrier family 6 (neurotransmitter transporter, dopamine), member 3
GRID2IP	0.98	0	392862	7	7p22.1	glutamate receptor, ionotropic, delta 2 (Grid2) interacting protein
PAX7	0.98	0	5081	1	1p36.13	paired box 7
ZNF536	0.98	0	9745	19	19q12	zinc finger protein 536
EN1	0.98	0	2019	2	2q14.2	engrailed homeobox 1
TBR1	0.979	0	10716	2	2q24	T-box, brain, 1
FLI1	0.979	0	2313	11	11q24.1-q24.3	Friend leukemia virus integration 1
CACNG3	0.979	0	10368	16	16p12.1	calcium channel, voltage-dependent, gamma subunit 3
GCM2	0.979	0	9247	6	6p23	glial cells missing homolog 2 (Drosophila)
PTPRR	0.979	0	5801	12	12q15	protein tyrosine phosphatase, receptor type, R
SEZ6	0.979	0	124925	17	17q11.2	seizure related 6 homolog (mouse)
CNGA3	0.979	0	1261	2	2q11.2	cyclic nucleotide gated channel alpha 3
VIPR2	0.979	0	7434	7	7q36.3	vasoactive intestinal peptide receptor 2
OTX2	0.979	0	5015	14	14q22.3	orthodenticle homeobox 2
LHX3	0.979	0	8022	9	9q34.3	LIM homeobox 3
DAB1	0.979	0	1600	1	1p32-p31	disabled homolog 1 (Drosophila)
RYR1	0.979	0	6261	19	19q13.1	ryanodine receptor 1 (skeletal)
DLG2	0.979	0	1740	11	11q14.1	discs, large homolog 2 (Drosophila)
FOXG1	0.979	0	2290	14	14q13	forkhead box G1
NTSR1	0.978	0	4923	20	20q13	neurotensin receptor 1 (high affinity)
SRRM4	0.978	0	84530	12	12q24.23	serine/arginine repetitive matrix 4
EBF2	0.978	0	64641	8	8p21.2	early B-cell factor 2
PRDM14	0.978	0	63978	8	8q13.3	PR domain containing 14
DLK1	0.978	0	8788	14	14q32	delta-like 1 homolog (Drosophila)
ANKS1B	0.978	0	56899	12	12q23.1	ankyrin repeat and sterile alpha motif domain containing 1B
EBF1	0.978	0	1879	5	5q34	early B-cell factor 1
BRSK2	0.978	0	9024	11	11p15.5	BR serine/threonine kinase 2
CELF5	0.978	0	60680	19	19p13	CUGBP, Elav-like family member 5
SLC12A5	0.978	0	57468	20	20q13.12	solute carrier family 12 (potassium/chloride transporter), member 5
HS3ST4	0.978	0	9951	16	16p11.2	heparan sulfate (glucosamine) 3-O-sulfotransferase 4
C1orf94	0.978	0	84970	1	1p34.3	chromosome 1 open reading frame 94
GDF6	0.978	0	392255	8	8q22.1	growth differentiation factor 6
RNF220	0.978	0	55182	1	1p34.1	ring finger protein 220
CPEB1	0.978	0	64506	15	15q25.2	cytoplasmic polyadenylation element binding protein 1
OTP	0.978	0	23440	5	5q13.3	orthopedia homeobox
DPP10	0.978	0	57628	2	2q14.1	dipeptidyl-peptidase 10 (non-functional)
GPR123	0.978	0	84435	10	10q26	G protein-coupled receptor 123
XKR4	0.978	0	114786	8	8q12.1	XK, Kell blood group complex subunit-related family, member 4
MAST1	0.978	0	22983	19	19p13.2	microtubule associated serine/threonine kinase 1
ALX4	0.978	0	60529	11	11p11.2	ALX homeobox 4
ALX3	0.978	0	257	1	1p13.3	ALX homeobox 3
MMEL1	0.977	0	79258	1	1p36	membrane metallo-endopeptidase-like 1
FAM19A1	0.977	0	407738	3	3p14.1	family with sequence similarity 19 (chemokine (C-C motif)-like), member A1
TBX15	0.977	0	6913	1	1p11.1	T-box 15
IRF4	0.977	0	3662	6	6p25-p23	interferon regulatory factor 4
FAM135B	0.977	0	51059	8	8q24.23	family with sequence similarity 135, member B
NR2E1	0.977	0	7101	6	6q21	nuclear receptor subfamily 2, group E, member 1
OTX2OS1	0.977	0	100309464	14		Otx2 opposite strand transcript 1
GRM1	0.977	0	2911	6	6q24	glutamate receptor, metabotropic 1
HAS1	0.977	0	3036	19	19q13.4	hyaluronan synthase 1
LMX1A	0.977	0	4009	1	1q24.1	LIM homeobox transcription factor 1, alpha
MMP9	0.977	0	4318	20	20q11.2-q13.1	matrix metallopeptidase 9 (gelatinase B, 92kDa gelatinase, 92kDa type IV collagenase)
UNC13A	0.977	0	23025	19	19p13.11	unc-13 homolog A (C. elegans)
CSMD1	0.977	0	64478	8	8p23.2	CUB and Sushi multiple domains 1
CHRM2	0.977	0	1129	7	7q31-q35	cholinergic receptor, muscarinic 2
GRM5	0.977	0	2915	11	11q14.3	glutamate receptor, metabotropic 5
FGF14	0.977	0	2259	13	13q34	fibroblast growth factor 14
PIK3R5	0.977	0	23533	17	17p13.1	phosphoinositide-3-kinase, regulatory subunit 5
LHX8	0.977	0	431707	1	1p31.1	LIM homeobox 8
NCAN	0.977	0	1463	19	19p12	neurocan
NELL1	0.977	0	4745	11	11p15.1	NEL-like 1 (chicken)
PRDM13	0.977	0	59336	6	6q16-q21	PR domain containing 13
LHFPL3	0.977	0	375612	7	7q22.2	lipoma HMGIC fusion partner-like 3
KIF19	0.977	0	124602	17	17q25.1	kinesin family member 19

Table S1: Meta-analysis: 100 most significant most unstable genes
