# Supplementary material for: Corruption of the Intra-Gene DNA Methylation Architecture Is a Hallmark of Cancer
Source: PLoS One. 2013 Jul 16;8(7):e68285. doi: 10.1371/journal.pone.0068285 (PMC3712966; doi:10.1371/journal.pone.0068285)
Supplement: Table S2 — Meta-analysis: 100 most significant least unstable genes. (PDF) [file pone.0068285.s007.pdf]

symbol	mean AUC	adj-p	Entrez	chr	band info	gene name
IER2	0.534	1	9592	19	19p13.2	immediate early response 2
ZNF33B	0.543	1	7582	10	10q11.2	zinc finger protein 33B
TM2D1	0.545	1	83941	1	1p31.3	TM2 domain containing 1
EXOSC6	0.545	1	118460	16	16q22.1	exosome component 6
DAD1	0.546	1	1603	14	14q11.2	defender against cell death 1
CDK7	0.549	1	1022	5	5q12.1	cyclin-dependent kinase 7
SDR39U1	0.55	1	56948	14	14q12	short chain dehydrogenase/reductase family 39U, member 1
DHPS	0.55	1	1725	19	19p13.2	deoxyhypusine synthase
PRIM1	0.551	1	5557	12	12q13	primase, DNA, polypeptide 1 (49kDa)
FBXL19-AS1	0.551	1	283932	16	16p11.2	FBXL19 antisense RNA 1 (non-protein coding)
HSDL2	0.552	1	84263	9	9q32	hydroxysteroid dehydrogenase like 2
LOC145783	0.553	1	145783	15	15q21.3	uncharacterized LOC145783
DDX18	0.554	1	8886	2	2q14.1	DEAD (Asp-Glu-Ala-Asp) box polypeptide 18
C12orf73	0.556	1	728568	12	12q23.3	chromosome 12 open reading frame 73
ZNF619	0.557	1	285267	3	3p22.1	zinc finger protein 619
WDR73	0.558	1	84942	15	15q25.2	WD repeat domain 73
GOLGB1	0.559	1	2804	3	3q13	golgin B1
C2orf49	0.559	1	79074	2	2q12.1	chromosome 2 open reading frame 49
LARS	0.559	1	51520	5	5q32	leucyl-tRNA synthetase
USP8	0.56	1	9101	15	15q21.2	ubiquitin specific peptidase 8
SAMD1	0.562	1	90378	19	19p13.12	sterile alpha motif domain containing 1
CCDC132	0.562	1	55610	7	7q21.3	coiled-coil domain containing 132
MRPS16	0.562	1	51021	10	10q22.1	mitochondrial ribosomal protein S16
PSMC6	0.564	1	5706	14	14q22.1	proteasome (prosome, macropain) 26S subunit, ATPase, 6
RSL24D1	0.566	1	51187	15	15q21	ribosomal L24 domain containing 1
RPRD1A	0.566	1	55197	18	18q12.2	regulation of nuclear pre-mRNA domain containing 1A
CDCA8	0.566	1	55143	1	1p34.3	cell division cycle associated 8
CAT	0.567	1	847	11	11p13	catalase
KIAA0100	0.567	1	9703	17	17q11.2	KIAA0100
ALG10B	0.568	1	144245	12	12q12	asparagine-linked glycosylation 10, alpha-1,2-glucosyltransferase homolog B (yeast)
MED17	0.568	1	9440	11	11q14	mediator complex subunit 17
LEO1	0.568	1	123169	15	15q21.2	Leo1, Paf1/RNA polymerase II complex component, homolog (S. cerevisiae)
MIR760	0.568	1	100126348	1	1p22.1	microRNA 760
COPS3	0.569	1	8533	17	17p11.2	COP9 constitutive photomorphogenic homolog subunit 3 (Arabidopsis)
ALG1	0.569	1	56052	16	16p13.3	asparagine-linked glycosylation 1, beta-1,4-mannosyltransferase homolog (S. cerevisiae)
SSB	0.57	1	6741	2	2q31.1	Sjogren syndrome antigen B (autoantigen La)
SSSCA1	0.57	1	10534	11	11q13.1	Sjogren syndrome/scleroderma autoantigen 1
MRPS31	0.571	1	10240	13	13q14.11	mitochondrial ribosomal protein S31
SLC9B1	0.571	1	150159	4	4q24	solute carrier family 9, subfamily B (cation proton antiporter 2), member 1
ZBED5	0.572	1	58486	11	11p15.3	zinc finger, BED-type containing 5
METTL3	0.572	1	56339	14	14q11.1	methyltransferase like 3
TSFM	0.573	1	10102	12	12q14.1	Ts translation elongation factor, mitochondrial
THAP9	0.573	1	79725	4	4q21.22	THAP domain containing 9
SAE1	0.573	1	10055	19	19q13.32	SUMO1 activating enzyme subunit 1
FAM54A	0.573	1	113115	6	6q23.3	family with sequence similarity 54, member A
FASTKD1	0.574	1	79675	2	2q31	FAST kinase domains 1
ARL1	0.574	1	400	12	12q23.2	ADP-ribosylation factor-like 1
MED30	0.574	1	90390	8	8q24.11	mediator complex subunit 30
CCNG1	0.575	1	900	5	5q32-q34	cyclin G1
SCAND1	0.575	1	51282	20	20q11.1-q11.23	SCAN domain containing 1
C12orf45	0.575	1	121053	12	12q23.3	chromosome 12 open reading frame 45
RNPC3	0.576	1	55599	1	1p21	RNA-binding region (RNP1, RRM) containing 3
SLC30A6	0.577	1	55676	2	2p22.3	solute carrier family 30 (zinc transporter), member 6
FAM32A	0.577	1	26017	19	19pter-p13.3	family with sequence similarity 32, member A
DPY30	0.577	1	84661	2	2p22.3	dpy-30 homolog (C. elegans)
NUP35	0.577	1	129401	2	2q32.1	nucleoporin 35kDa
CATSPER2	0.577	1	117155	15	15q15.3	cation channel, sperm associated 2
ALG10	0.578	1	84920	12	12p11.1	asparagine-linked glycosylation 10, alpha-1,2-glucosyltransferase homolog (S. pombe)
MRPL34	0.578	1	64981	19	19p13.1	mitochondrial ribosomal protein L34
DPAGT1	0.579	1	1798	11	11q23.3	dolichyl-phosphate (UDP-N-acetylglucosamine) N-acetylglucosaminephosphotransferase 1 (
COX7A2L	0.579	1	9167	2	2p21	cytochrome c oxidase subunit VIIa polypeptide 2 like
MRPL11	0.579	1	65003	11	11q13.3	mitochondrial ribosomal protein L11
CDKN2AIPNL	0.579	1	91368	5	5q31.1	CDKN2A interacting protein N-terminal like
SAP30L	0.579	1	79685	5	5q33.2	SAP30-like
PSMD3	0.58	1	5709	17	17q21.1	proteasome (prosome, macropain) 26S subunit, non-ATPase, 3
SOS2	0.58	1	6655	14	14q21	son of sevenless homolog 2 (Drosophila)
WDR31	0.58	1	114987	9	9q32	WD repeat domain 31
C9orf93	0.581	1	203238	9	9p22.3	chromosome 9 open reading frame 93
LOC400027	0.581	1	400027	12	12q12	uncharacterized LOC400027
C18orf21	0.581	1	83608	18	18q12.2	chromosome 18 open reading frame 21
RNU11	0.581	1	26824	1	1p35	RNA, U11 small nuclear
PIGM	0.582	1	93183	1	1q23.2	phosphatidylinositol glycan anchor biosynthesis, class M
CREBZF	0.582	1	58487	11	11q14	CREB/ATF bZIP transcription factor
NUDT9	0.583	1	53343	4	4q22.1	nudix (nucleoside diphosphate linked moiety X)-type motif 9
AASDH	0.583	1	132949	4	4q12	aminoadipate-semialdehyde dehydrogenase
ARFGEF2	0.583	1	10564	20	20q13.13	ADP-ribosylation factor guanine nucleotide-exchange factor 2 (brefeldin A-inhibited)
USO1	0.583	1	8615	4	4q21.1	USO1 vesicle docking protein homolog (yeast)
FANCL	0.583	1	55120	2	2p16.1	Fanconi anemia, complementation group L
RPF1	0.583	1	80135	1	1p22.3	ribosome production factor 1 homolog (S. cerevisiae)
CCNB2	0.583	1	9133	15	15q22.2	cyclin B2
OCIAD1	0.583	1	54940	4	4p11	OCIA domain containing 1
HSD17B11	0.583	1	51170	4	4q22.1	hydroxysteroid (17-beta) dehydrogenase 11
C11orf65	0.584	1	160140	11	11q22.3	chromosome 11 open reading frame 65
ZNF226	0.584	1	7769	19	19q13.2	zinc finger protein 226
ROCK1	0.584	1	6093	18	18q11.1	Rho-associated, coiled-coil containing protein kinase 1
ZFAND5	0.584	1	7763	9	9q13-q21	zinc finger, AN1-type domain 5
UQCRC2	0.585	1	7385	16	16p12	ubiquinol-cytochrome c reductase core protein II
ALMS1	0.585	1	7840	2	2p13	Alstrom syndrome 1
LOC391322	0.585	1	391322	22	22q11.23	D-dopachrome tautomerase-like
TEFM	0.585	1	79736	17		transcription elongation factor, mitochondrial
ZWINT	0.585	1	11130	10	10q21-q22	ZW10 interactor
RPS27L	0.585	1	51065	15	15q22.2	ribosomal protein S27-like
FAM151B	0.585	1	167555	5	5q14.1	family with sequence similarity 151, member B
C12orf44	0.587	1	60673	12	12q13.13	chromosome 12 open reading frame 44
MSH2	0.587	1	4436	2	2p21	mutS homolog 2, colon cancer, nonpolyposis type 1 (E. coli)
PAPD4	0.587	1	167153	5	5q14.1	PAP associated domain containing 4
PPID	0.587	1	5481	4	4q31.3	peptidylprolyl isomerase D
ZRANB2	0.587	1	9406	1	1p31	zinc finger, RAN-binding domain containing 2
RPS21	0.587	1	6227	20	20q13.3	ribosomal protein S21
TWF1	0.588	1	5756	12	12q12	twinfilin, actin-binding protein, homolog 1 (Drosophila)

Table S2: Meta-analysis: 100 most significant least unstable genes
