# Supplementary material for: Corruption of the Intra-Gene DNA Methylation Architecture Is a Hallmark of Cancer
Source: PLoS One. 2013 Jul 16;8(7):e68285. doi: 10.1371/journal.pone.0068285 (PMC3712966; doi:10.1371/journal.pone.0068285)
Supplement: Table S3 — Meta-analysis GSEA: most unstable genes, 100 most significant gene-sets. (PDF) [file pone.0068285.s008.pdf]

gene set	OR	OR lower 95% CI	OR upper 95% CI	adj-p
BENPORATH_ES_WITH_H3K27ME3	7.37	6.37	8.54	1.31e-180
BENPORATH_SUZ12_TARGETS	6.8	5.84	7.94	6.79e-150
BENPORATH_EED_TARGETS	5.35	4.64	6.19	6.45e-122
BENPORATH_PRC2_TARGETS	7.98	6.57	9.74	7.05e-115
CAGGTG_V$E12_Q6	2.21	1.98	2.47	8.81e-43
PLASMA_MEMBRANE	2.32	2.04	2.63	2.75e-36
ANATOMICAL_STRUCTURE_DEVELOPMENT	2.54	2.2	2.92	1.54e-34
AACTTT_UNKNOWN	2.21	1.96	2.5	5.88e-34
MULTICELLULAR_ORGANISMAL_DEVELOPMENT	2.45	2.13	2.83	1.42e-32
PLASMA_MEMBRANE_PART	2.37	2.07	2.72	5.74e-32
SYSTEM_DEVELOPMENT	2.62	2.24	3.06	6.4e-32
module_100	3.25	2.67	3.95	1.14e-30
module_137	3.23	2.66	3.93	2.12e-30
module_66	3.19	2.63	3.88	2.65e-30
SYSTEM_PROCESS	3.18	2.62	3.85	3.18e-30
HATADA_METHYLATED_IN_LUNG_CANCER_UP	3.98	3.14	5.05	7.84e-30
INTRINSIC_TO_PLASMA_MEMBRANE	2.41	2.08	2.8	1.03e-28
INTEGRAL_TO_PLASMA_MEMBRANE	2.4	2.07	2.79	6.78e-28
REACTOME_DOWNSTREAM_EVENTS_IN_GPCR_SIGNALING	3.46	2.78	4.32	1.85e-27
module_11	2.97	2.44	3.6	1.93e-26
KEGG_NEUROACTIVE_LIGAND_RECEPTOR_INTERACTION	4.56	3.44	6.08	7.33e-26
NEUROLOGICAL_SYSTEM_PROCESS	3.63	2.87	4.61	9.82e-26
INTRINSIC_TO_MEMBRANE	2.03	1.79	2.31	1.3e-24
INTEGRAL_TO_MEMBRANE	2.03	1.78	2.31	5.49e-24
MEMBRANE	1.82	1.63	2.03	6.36e-24
NERVOUS_SYSTEM_DEVELOPMENT	3.31	2.63	4.16	2.87e-23
GGGAGGRR_V$MAZ_Q6	1.82	1.63	2.04	1.18e-22
REACTOME_GPCR_LIGAND_BINDING	3.35	2.64	4.26	8.15e-22
SYNAPTIC_TRANSMISSION	5.37	3.79	7.7	9.72e-22
MEMBRANE_PART	1.84	1.64	2.07	1.04e-21
TAATTA_V$CHX10_01	2.54	2.12	3.05	1.54e-21
RECEPTOR_ACTIVITY	2.65	2.19	3.22	1.94e-21
TRANSMISSION_OF_NERVE_IMPULSE	4.9	3.53	6.87	2.06e-21
TGGAAA_V$NFAT_Q4_01	1.91	1.68	2.17	4.3e-21
CELL_CELL_SIGNALING	3.07	2.45	3.85	6.07e-21
TRANSMEMBRANE_RECEPTOR_ACTIVITY	3.03	2.42	3.8	1.46e-20
module_220	3.28	2.57	4.19	1.94e-20
CAGCTG_V$AP4_Q5	1.97	1.71	2.26	1.59e-19
ION_TRANSMEMBRANE_TRANSPORTER_ACTIVITY	3.57	2.71	4.71	1.17e-18
LIU_PROSTATE_CANCER_DN	2.65	2.15	3.26	1.63e-18
ION_CHANNEL_ACTIVITY	5.46	3.72	8.13	1.87e-18
G_PROTEIN_COUPLED_RECEPTOR_PROTEIN_SIGNALING_PATHW	3.22	2.49	4.16	4.54e-18
SUBSTRATE_SPECIFIC_CHANNEL_ACTIVITY	5.09	3.51	7.47	7.05e-18
CTTTGT_V$LEF1_Q2	1.75	1.55	1.98	8.74e-17
GATED_CHANNEL_ACTIVITY	5.84	3.82	9.09	1.12e-16
KEGG_CALCIUM_SIGNALING_PATHWAY	4.26	3.04	6.02	1.71e-16
G_PROTEIN_COUPLED_RECEPTOR_ACTIVITY	4.54	3.18	6.55	2.12e-16
TGATTTRY_V$GFI1_01	3.6	2.68	4.86	2.77e-16
CATION_CHANNEL_ACTIVITY	5.68	3.71	8.86	6.34e-16
CELL_SURFACE_RECEPTOR_LINKED_SIGNAL_TRANSDUCTION_G	2.22	1.85	2.67	1.71e-15
ACEVEDO_METHYLATED_IN_LIVER_CANCER_DN	2.12	1.78	2.52	1.72e-15
SUBSTRATE_SPECIFIC_TRANSMEMBRANE_TRANSPORTER_ACTIV	2.84	2.22	3.63	1.98e-15
METAL_ION_TRANSMEMBRANE_TRANSPORTER_ACTIVITY	4.68	3.21	6.91	2.27e-15
GCANCTGNY_V$MYOD_Q6	2.13	1.78	2.53	2.59e-15
ORGAN_DEVELOPMENT	2.24	1.85	2.7	4.2e-15
CTTTGA_V$LEF1_Q2	1.93	1.65	2.26	9.55e-15
YCATTAA_UNKNOWN	2.52	2.02	3.14	1.1e-14
SCHUETZ_BREAST_CANCER_DUCTAL_INVASIVE_UP	2.76	2.16	3.52	1.11e-14
V$GATA1_04	3.94	2.81	5.57	1.11e-14
CATION_TRANSMEMBRANE_TRANSPORTER_ACTIVITY	3.55	2.6	4.87	1.65e-14
TRANSMEMBRANE_TRANSPORTER_ACTIVITY	2.62	2.08	3.31	2.23e-14
TTGTTT_V$FOXO4_01	1.66	1.47	1.88	6.94e-14
SECOND_MESSENGER_MEDIATED_SIGNALING	4.35	2.98	6.42	1.04e-13
TTANTCA_UNKNOWN	2.03	1.7	2.41	1.33e-13
module_27	2.68	2.09	3.43	1.47e-13
V$NRSF_01	7.39	4.25	13.4	1.9e-13
module_64	2.29	1.86	2.81	2.05e-13
module_12	2.55	2.02	3.23	2.64e-13
SMID_BREAST_CANCER_NORMAL_LIKE_UP	2.35	1.89	2.91	3.04e-13
CAGGTA_V$AREB6_01	2.09	1.73	2.53	1.57e-12
REACTOME_CLASS_A1_RHODOPSIN_LIKE_RECEPTORS	2.95	2.22	3.92	1.91e-12
METAL_ION_TRANSPORT	4.7	3.08	7.27	1.97e-12
V$CDC5_01	3.31	2.41	4.57	2.53e-12
TGACATY_UNKNOWN	2.16	1.76	2.63	2.53e-12
SUBSTRATE_SPECIFIC_TRANSPORTER_ACTIVITY	2.4	1.91	3.01	2.55e-12
module_274	6.33	3.74	11.1	2.94e-12
V$LHX3_01	3.49	2.49	4.91	3.5e-12
RIGGI_EWING_SARCOMA_PROGENITOR_UP	2.31	1.85	2.87	3.63e-12
module_47	3.06	2.26	4.14	5.97e-12
ANATOMICAL_STRUCTURE_MORPHOGENESIS	2.36	1.88	2.96	6.33e-12
ION_TRANSPORT	3.36	2.42	4.69	6.33e-12
V$OCT1_05	3.29	2.38	4.57	6.86e-12
YATTNATC_UNKNOWN	2.77	2.1	3.64	6.86e-12
TGACAGNY_V$MEIS1_01	1.99	1.66	2.39	8.07e-12
YATGNWAAT_V$OCT_C	2.77	2.1	3.65	8.08e-12
GGATTA_V$PITX2_Q2	2.22	1.8	2.75	8.08e-12
module_117	1.99	1.66	2.39	8.08e-12
CATION_TRANSPORT	3.82	2.64	5.57	8.08e-12
module_41	2.08	1.71	2.53	1.06e-11
VOLTAGE_GATED_CHANNEL_ACTIVITY	6.45	3.72	11.6	1.71e-11
G_PROTEIN_SIGNALING__COUPLED_TO_CYCLIC_NUCLEOTIDE_	5.03	3.16	8.17	2.01e-11
SHEN_SMARCA2_TARGETS_DN	2.56	1.98	3.31	2.15e-11
RHODOPSIN_LIKE_RECEPTOR_ACTIVITY	4.53	2.94	7.08	2.15e-11
REACTOME_NCAM1_INTERACTIONS	14	5.79	40.9	2.67e-11
YAUCH_HEDGEHOG_SIGNALING_PARACRINE_DN	2.92	2.17	3.93	2.94e-11
V$HNF6_Q6	3.31	2.36	4.64	3.27e-11
RTAAACA_V$FREAC2_01	1.87	1.58	2.22	3.27e-11
CYCLIC_NUCLEOTIDE_MEDIATED_SIGNALING	4.86	3.07	7.85	3.93e-11
VOLTAGE_GATED_CATION_CHANNEL_ACTIVITY	6.76	3.79	12.6	4.09e-11
LEE_NEURAL_CREST_STEM_CELL_UP	3.76	2.58	5.54	4.66e-11

Table S3: Meta-analysis GSEA: most unstable genes, 100 most significant gene-sets
