# Supplementary material for: Corruption of the Intra-Gene DNA Methylation Architecture Is a Hallmark of Cancer
Source: PLoS One. 2013 Jul 16;8(7):e68285. doi: 10.1371/journal.pone.0068285 (PMC3712966; doi:10.1371/journal.pone.0068285)
Supplement: Table S4 — Meta-analysis GSEA: least unstable genes, 100 most significant gene-sets. (PDF) [file pone.0068285.s009.pdf]

gene set	OR	OR lower 95% CI	OR upper 95% CI	adj-p
DODD_NASOPHARYNGEAL_CARCINOMA_DN	2.7	2.33	3.11	3.13e-35
DIAZ_CHRONIC_MEYLOGENOUS_LEUKEMIA_UP	2.53	2.2	2.9	1.34e-34
PUJANA_BRCA1_PCC_NETWORK	2.39	2.1	2.73	2.91e-33
PUJANA_CHEK2_PCC_NETWORK	3.1	2.59	3.7	1.04e-29
RODRIGUES_THYROID_CARCINOMA_POORLY_DIFFERENTIATED_	3.34	2.73	4.06	7.09e-28
GARY_CD5_TARGETS_DN	4.07	3.22	5.14	1.72e-27
KINSEY_TARGETS_OF_EWSR1_FLII_FUSION_UP	2.3	1.99	2.67	4.43e-24
NUCLEUS	2.17	1.88	2.49	2.03e-22
MILI_PSEUDOPODIA_HAPTOTAXIS_UP	3.45	2.74	4.33	2.32e-22
ACEVEDO_LIVER_TUMOR_VS_NORMAL_ADJACENT_TISSUE_UP	2.63	2.2	3.15	4.16e-22
ORGANELLE_PART	2.26	1.94	2.63	1.81e-21
INTRACELLULAR_ORGANELLE_PART	2.26	1.93	2.63	2.12e-21
RODRIGUES_THYROID_CARCINOMA_ANAPLASTIC_UP	2.68	2.21	3.25	4.87e-20
SHEN_SMARCA2_TARGETS_UP	3.34	2.63	4.23	1.74e-19
TIEN_INTESTINE_PROBIOTICS_24HR_UP	2.85	2.31	3.51	3.86e-19
SCGGAAGY_V$ELK1_02	2.52	2.09	3.02	4.31e-19
ZHANG_BREAST_CANCER_PROGENITORS_UP	3.35	2.62	4.28	9.44e-19
module_83	4.04	3.04	5.36	1.02e-18
BENPORATH_MYC_MAX_TARGETS	2.52	2.09	3.03	1.23e-18
CAIRO_HEPATOBLASTOMA_CLASSES_UP	2.71	2.21	3.32	3.58e-18
ACEVEDO_LIVER_CANCER_UP	2.3	1.93	2.72	3.74e-18
module_114	3.66	2.78	4.8	2.75e-17
NUCLEAR_PART	2.74	2.21	3.39	5.06e-17
module_151	3.7	2.79	4.89	7.36e-17
BLALOCK_ALZHEIMERS_DISEASE_DN	1.99	1.71	2.31	2.37e-16
KRIGE_RESPONSE_TO_TOSEDOSTAT_24HR_DN	2.14	1.81	2.53	6.9e-16
MORF_RAN	3.89	2.85	5.29	5.37e-15
MORF_SOD1	3.74	2.76	5.04	7.07e-15
ORGANELLE_LUMEN	2.81	2.22	3.56	1.51e-14
MEMBRANE_ENCLOSED_LUMEN	2.81	2.22	3.56	1.51e-14
WONG_MITOCHONDRIA_GENE_MODULE	4.26	3.03	5.97	1.85e-14
WEI_MYCN_TARGETS_WITH_E_BOX	2.24	1.85	2.69	2.63e-14
REACTOME_CELL_CYCLE_MITOTIC	3.19	2.43	4.19	7.2e-14
DANG_BOUND_BY_MYC	2	1.7	2.36	8.71e-14
OSMAN_BLADDER_CANCER_UP	2.8	2.2	3.57	8.71e-14
REACTOME_GENE_EXPRESSION	2.73	2.13	3.48	1.04e-12
GNF2_DENR	23.7	8.91	79.3	1.19e-12
CHEN_HOXA5_TARGETS_9HR_UP	3.41	2.51	4.61	1.52e-12
RAMALHO_STEMNESS_UP	3.69	2.66	5.11	2.09e-12
SCHLOSSER_SERUM_RESPONSE_DN	2.15	1.77	2.6	2.16e-12
MOOTHA_MITOCHONDRIA	2.63	2.06	3.33	2.17e-12
NUCLEAR_LUMEN	2.8	2.16	3.61	2.49e-12
MORF_RAD23B	4.34	2.98	6.3	3.02e-12
WANG_LMO4_TARGETS_DN	2.81	2.16	3.63	3.73e-12
WONG_EMBRYONIC_STEM_CELL_CORE	2.94	2.23	3.84	4.18e-12
MORF_RAB1A	4.01	2.81	5.72	4.18e-12
MOOTHA_HUMAN_MITODB_6_2002	2.6	2.04	3.31	4.82e-12
REACTOME_MITOTIC_M_M_G1_PHASES	4.33	2.96	6.31	4.82e-12
WINNEPENNINCKX_MELANOMA_METASTASIS_UP	4.45	3.01	6.58	1.01e-11
NOUZOVA_TRETINOIN_AND_H4_ACETYLATION	5.23	3.36	8.16	1.61e-11
REACTOME_CELL_CYCLE_CHECKPOINTS	5.37	3.42	8.45	1.61e-11
MACROMOLECULAR_COMPLEX	1.93	1.62	2.29	3.61e-11
SPIELMAN_LYMPHOBLAST_EUROPEAN_VS_ASIAN_DN	2.2	1.78	2.7	4.36e-11
KRIGE_RESPONSE_TO_TOSEDOSTAT_6HR_DN	1.95	1.63	2.33	6.57e-11
NUYTTEN_EZH2_TARGETS_DN	1.92	1.61	2.29	1.28e-10
HORIUCHI_WTAP_TARGETS_DN	2.87	2.15	3.81	1.36e-10
MORF_PSMC2	5.6	3.45	9.15	1.36e-10
MORF_PSMC1	3.92	2.69	5.7	1.6e-10
MORF_RAD21	3.92	2.69	5.7	1.6e-10
MORF_DAP3	3.79	2.62	5.45	1.73e-10
REACTOME_INFLUENZA_LIFE_CYCLE	4.67	3.03	7.18	1.79e-10
MORF_SKP1A	3.7	2.57	5.3	2.11e-10
REACTOME_REGULATION_OF_APC_ACTIVATORS_BETWEEN_G1_S	6.4	3.74	11.1	3.09e-10
MOREAUX_MULTIPLE_MYELOMA_BY_TACI_DN	4.88	3.11	7.67	3.42e-10
ENK_UV_RESPONSE_KERATINOCYTE_DN	2.22	1.77	2.77	5e-10
REACTOME_AUTODEGRADATION_OF_CDH1_BY_CDH1_APC	7.92	4.27	15.1	5e-10
REACTOME_CDC20_PHOSPHO_APC_MEDIATED_DEGRADATION_OF	6.91	3.89	12.5	7.41e-10
MORF_NME2	4.1	2.73	6.13	7.41e-10
NUCLEOPLASM	2.86	2.12	3.84	7.46e-10
TARTE_PLASMA_CELL_VS_PLASMABLAST_DN	2.71	2.04	3.57	7.74e-10
STARK_PREFRONTAL_CORTEX_22Q11_DELETION_DN	2.37	1.85	3.01	8.08e-10
MANALO_HYPOXIA_DN	2.85	2.11	3.82	1.18e-09
BENPORATH_CYCLING_GENES	2.03	1.66	2.49	1.24e-09
PUJANA_XPRSS_INT_NETWORK	3.72	2.54	5.42	1.28e-09
KIM_WT1_TARGETS_DN	2.27	1.79	2.86	1.49e-09
GCM_ACTG1	4.65	2.94	7.34	2.45e-09
PUJANA_BRCA2_PCC_NETWORK	2.32	1.81	2.95	2.75e-09
MORF_HDAC2	2.81	2.08	3.79	2.96e-09
REACTOME_VIF_MEDIATED_DEGRADATION_OF_APOBEC3G	8.81	4.43	18.3	3.04e-09
module_98	2.35	1.82	3.01	3.59e-09
MORF_PPP1CA	3.88	2.59	5.79	3.82e-09
GNF2_APEX1	6.08	3.49	10.7	4.5e-09
GCM_CSNK2B	5.37	3.21	9.02	4.92e-09
module_252	2.86	2.09	3.9	5.06e-09
REACTOME_METABOLISM_OF_PROTEINS	3.26	2.29	4.62	5.51e-09
module_32	3.03	2.17	4.2	5.64e-09
BERENJENO_TRANSFORMED_BY_RHOA_UP	2.12	1.69	2.64	6.99e-09
MORF_UBE2I	3.1	2.2	4.34	7.52e-09
NUCLEOBASE__NUCLEOSIDE__NUCLEOTIDE_AND_NUCLEIC_ACI	1.68	1.43	1.96	7.52e-09
GNF2_DAP3	5.2	3.12	8.69	7.9e-09
RNA_PROCESSING	3.8	2.53	5.68	8.87e-09
RNA_BINDING	2.89	2.09	3.96	8.94e-09
REACTOME_STABILIZATION_OF_P53	8.47	4.23	17.7	9.7e-09
SHEDDEN_LUNG_CANCER_POOR_SURVIVAL_A6	2.21	1.73	2.8	1.25e-08
MORF_SP3	5.89	3.36	10.4	1.25e-08
GNF2_RBBP6	7.71	3.98	15.4	1.25e-08
GEORGES_TARGETS_OF_MIR192_AND_MIR215	1.82	1.51	2.17	1.38e-08
RHEIN_ALL_GLUCOCORTICOID_THERAPY_DN	2.39	1.83	3.1	1.61e-08
REACTOME_HIV_INFECTION	3.11	2.19	4.4	1.73e-08
MORF_RAC1	3.06	2.16	4.31	1.89e-08

Table S4: Meta-analysis GSEA: least unstable genes, 100 most significant gene-sets
